# Potential of Venom-Derived Compounds for the Development of New Antimicrobial Agents

**DOI:** 10.3390/toxins17050238

**Published:** 2025-05-11

**Authors:** Esraa Yasser Rabea, Esraa Dakrory Mahmoud, Nada Khaled Mohamed, Erada Rabea Ansary, Mahmoud Roushdy Alrouby, Rabab Reda Shehata, Youssef Yasser Mokhtar, Prakash Arullampalam, Ahmed M. Hegazy, Ahmed Al-Sabi, Tarek Mohamed Abd El-Aziz

**Affiliations:** 1 Biochemistry Division, Chemistry Department, Faculty of Science, Minia University, El-Minia 61519, Egypt; 81001148@sci.s-mu.edu.eg (E.Y.R.); 81000340@sci.s-mu.edu.eg (E.D.M.); 81044477@sci.s-mu.edu.eg (N.K.M.); 80999431@sci.s-mu.edu.eg (E.R.A.); 81035892@sci.s-mu.edu.eg (M.R.A.); 81014278@sci.s-mu.edu.eg (R.R.S.); 81052355@sci.s-mu.edu.eg (Y.Y.M.); 2 Department of Internal Medicine, Cardiovascular Division, Washington University School of Medicine, St. Louis, MO 63110, USA; aprakash@wustl.edu; 3 Zoology Department, Faculty of Science, Minia University, El-Minia 61519, Egypt; hegazy@mu.edu.eg; 4 College of Integrative Studies, Abdullah Al Salem University, Khaldiya 72303, Kuwait

**Keywords:** natural products, animal venoms, drug discovery, antimicrobial peptides, antibiotics, multi-drug resistant

## Abstract

The emergence of antimicrobial resistance is a significant challenge in global healthcare, necessitating innovative techniques to address multidrug-resistant pathogens. Multidrug-resistant pathogens like *Klebsiella pneumoniae*, *Acinetobacter baumannii*, and *Pseudomonas aeruginosa* pose significant public health threats, as they are increasingly resistant to common antibiotics, leading to more severe and difficult-to-treat infections. These pathogens are part of the ESKAPE group, which includes *Enterococcus faecium*, *Staphylococcus aureus*, and *Enterobacter* species. Animal venoms, derived from a wide range of species such as snakes, scorpions, spiders, bees, wasps, and ants, represent a rich source of bioactive peptides. Venoms have been a valuable source for drug discovery, providing unique compounds with therapeutic potential. Venom-derived drugs are known for their increased bioactivity, specificity, and stability compared to synthetic alternatives. These compounds are being investigated for various conditions, including treatments for diabetes, pain relief, cancer, and infections, showcasing their remarkable antimicrobial efficacy. In this review, we provide a comprehensive investigation into the potential of venom-derived compounds for developing new antimicrobial agents, including antibacterial, antifungal, antiviral, and antiparasitic therapeutics. Key venom components, including melittin from bee venom, phospholipase A_2_ from snake venom, and chlorotoxin from scorpion venom, exhibit potent antimicrobial effects through mechanisms such as membrane disruption, enzymatic inhibition, and immune modulation. We also explore the challenges related to the development and clinical use of venom-derived antimicrobials, including toxicity, stability, and delivery mechanisms. These compounds hold immense promise as transformative tools against resistant pathogens, offering a unique avenue for groundbreaking advancements in antimicrobial research and therapeutic development.

## 1. Introduction

Infectious diseases have garnered unprecedented attention, owing to the escalating challenge of their treatment, which has become increasingly complex and, at times, impossible [1]. Historically, antibiotics have formed the cornerstone of infectious disease management. However, the widespread and often misguided application of certain conventional antibiotics has triggered a crisis of antimicrobial resistance, leaving once-reliable therapies powerless and creating a grave public health emergency. The World Health Organization (WHO) has identified antimicrobial resistance (AMR) as one of the top ten global public health threats to humanity [2,3]. It is estimated that bacterial AMR was directly responsible for 1.27 million global deaths in 2019 and contributed to 4.95 million deaths [3]. Additionally, the COVID-19 pandemic has further emphasized the urgency of addressing infectious diseases [4]. Genomic analyses of bacterial strains have revealed over 20,000 genes implicated in multi-drug resistance (MDR), accentuating the relentless evolution of drug-resistance mechanisms across diverse pathogens [5]. The widespread impact of MDR pathogens manifests in a spectrum of debilitating illnesses, ranging from bacteremia and hepatitis induced by Gram-negative *Burkholderia pseudomallei* to skin infections, toxic shock syndrome, psoriasis, and pneumonia instigated by Gram-positive *Staphylococcus aureus* [6,7,8,9]. The extreme implications of this public health crisis necessitate the urgent pursuit of innovative approaches and efficacious strategies to combat MDR pathogens.

Natural products, derived from various biological sources, offer a rich reservoir of potentially novel drugs to combat the growing threat of drug-resistant infections. Plant extracts, algae, and animal venoms have attracted global attention for their rich content of bio-active compounds with a variety of biological effects, including antimicrobial, cytotoxic, hemolytic, and anti-inflammatory properties [10,11,12]. These natural products are more accessible and cost-effective than synthetic chemicals, which are expensive, toxic, and adversely affect healthy cells. Among these natural sources, animal venom stands out as a particularly powerful tool, not only for its biological richness, but also for its evolutionary role in survival. Animal venom is a natural defense mechanism enabling animals to subdue prey or deter predators. Interestingly, animal venoms also exhibit significant antimicrobial properties against a broad spectrum of pathogens, including bacteria, fungi, viruses, and parasites [13,14]. For instance, L-amino acid oxidase (L-AAO) from *Bothrops marajoensis* snake venom has been shown to combat fungi (*Candida albicans*), bacteria (*S. aureus*), and parasites (*Leishmania chagasi* and *Leishmania amazonensis*) [15].

**Figure 1:**
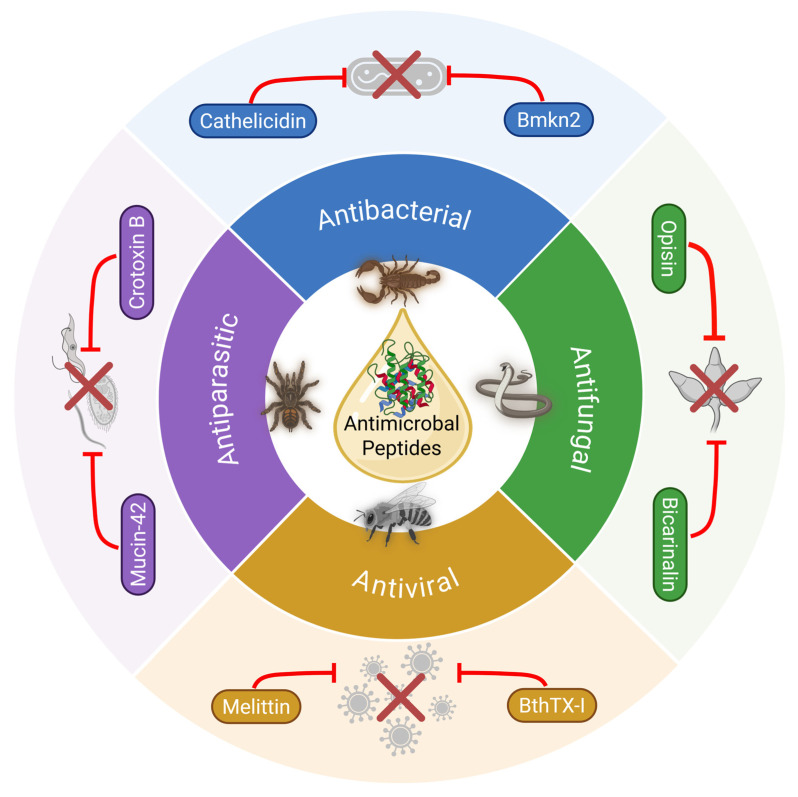
Schematic representation of animal venoms reviewed for their antimicrobial properties, including antibacterial, antifungal, antiviral, and antiparasitic activities. Created in BioRender. Abd El-Aziz, T.M. 2025. https://BioRender.com.

Animal venom is a complex mixture of bioactive substances, including polypeptides, enzymes, proteins, hydrocarbons, alkaloids, free amino acids, biogenic amines, formic acid, and other components [16]. Peptides purified from animal venom are particularly noted for their anti-microbial activity (Figure 1). Characteristics such as charge, hydrophobicity, and structural stereochemistry make these peptides highly specific to their targets, causing minimal damage to normal cells while exhibiting a broad spectrum of antimicrobial activity. Specific peptides derived from *Polistes wattii cameron* wasp venom demonstrate antimicrobial activity against four strains of multi-drug-resistant bacteria: *S. aureus*, *Streptococcus mutans*, *Salmonella typhimurium*, and *Enterobacter cloacae* [17]. Venom-derived antimicrobial agents are exciting due to their multiple mechanisms to overcome microbial resistance [18]. These mechanisms involve the formation of pores in microbial membranes, leading to the release of cytoplasmic contents and eventual cell death. Additionally, they down-regulate phosphorylation processes critical for protein synthesis signaling pathways, disrupt cell walls, inhibit bacterial growth, and interfere with lipid bilayers, ultimately resulting in either cell apoptosis or necrosis [18,19,20]. This diverse array of actions significantly reduces the likelihood of microbes developing resistance to these agents.

This review aims to build upon and extend previous work by providing a broad yet detailed perspective on venom-derived antimicrobial compounds, focusing on their diverse mechanisms of action, structural properties, and therapeutic potential. Unlike prior reviews, we place special emphasis on recent advances in drug development strategies, including chemical modifications, nanocarrier-based delivery, and bioengineering approaches that enhance the stability, specificity, and clinical applicability of venom-derived molecules. By integrating fundamental discoveries with translational insights, this review provides a forward-looking perspective on how venom-based therapeutics can be developed into viable antimicrobial agents, addressing key challenges and opportunities in this rapidly evolving field.

## 2. Venomous Animals as a Source of Antimicrobial Compounds

Venomous animals have long been identified as a rich source of bioactive compounds with various biological activities [11,12]. Currently, several prominent drugs derived from animal venoms have been approved by the US Food and Drug Administration (FDA) for human use, with others either undergoing or progressing through clinical trials (Table 1). A leading example of such drugs is Captopril (Capoten^®^, Bristol-Myers Squibb, New York City, NY, USA), an antihypertensive drug developed based on the bradykinin potentiating factor (BPF) found in the venom of the Brazilian pit viper, *Bothrops jararaca* [21]. Captopril, approved in 1981, was the first drug derived from an animal toxin to be approved for human use. Another example is Tirofiban, the first venom-derived anti-platelet drug, based on the structure of echistatin. It is a peptide extracted from the venom of the saw-scaled viper, *Echis carinatus* [22]. Additionally, Eptifibatide is a platelet aggregation inhibitor used to prevent blood clots, derived from the venom of the southeastern pygmy rattlesnake, *Sistrurus miliarius barbouri* [23]. Furthermore, Batroxobin, derived from the venom of the Brazilian lancehead pit viper (*Bothrops moojeni*), is used to treat thrombotic disorders [24]. Another notable drug is Exenatide, a medication for type 2 diabetes derived from the venom of the Gila monster, *Heloderma suspectum* [25]. From the cone snail’s venom, *Conus magus*, ziconotide, is a potent analgesic approved in 2004 for treating chronic pain [26]. These drugs demonstrate the innovative use of venom components in advanced medicine, highlighting the potential of natural toxins to resolve complex medical conditions.

Antimicrobial compounds from venom have gathered significant attention for their potential therapeutic applications [14]. From snakes and scorpions to spiders, bees, wasps, and ants, a wide array of venomous animals produces compounds capable of combating microbial pathogens. These compounds, from peptides and proteins to enzymes and small molecules, exhibit noteworthy anti-microbial properties against bacteria, fungi, viruses, and parasites [13]. This section investigates the attractive world of venom-derived antimicrobial compounds, exploring their therapeutic potential across various venomous animal species (Table 2).

### 2.1. Antimicrobial Agents from Snakes

**Figure 2:**
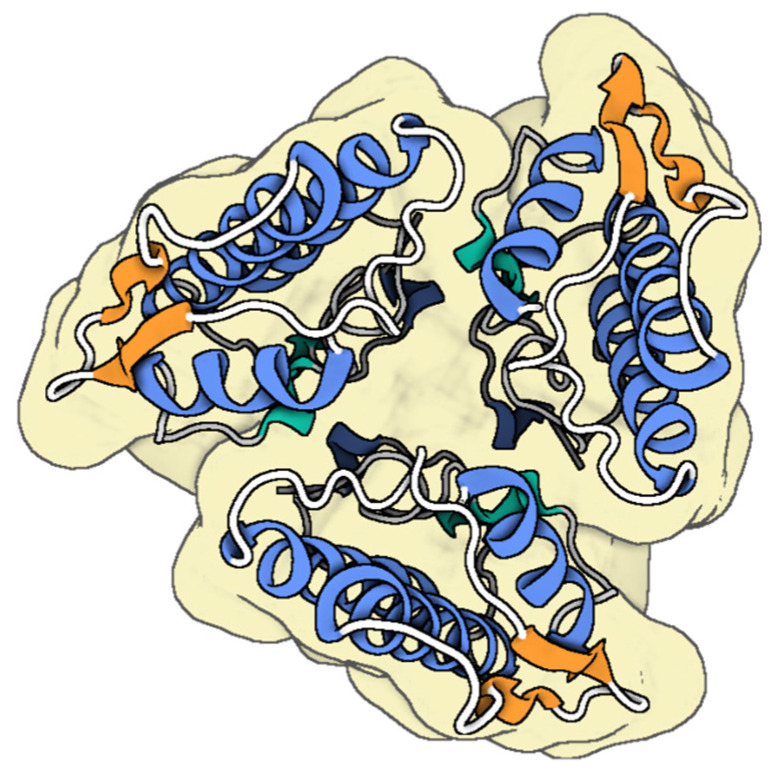
The molecular structure PLA_2_ from *Agkistrodon halys* [77]. The colored parts reflect the secondary conformation structures of the peptides (the blue for the helices, orange for the β-sheets, hinges in cyan, and loops in gray). The molecular structure was visualized by BioRender. Al-Sabi, A. 2025. https://BioRender.com.

Snake venoms represent a valuable source of biologically active substances, including oligopeptides such as waprins and larger polypeptides (e.g., cardiotoxins), along with proteins such as lectins, metalloproteinases, serine proteinases, L-amino acid oxidases, and phospholipases type A_2_ (PLA_2_; Figure 2) [74]. Numerous in vitro studies have demonstrated the bactericidal and bacteriostatic activities of snake venom PLA_2_s against Gram-positive bacteria, such as *S. aureus* and *Bacillus subtilis*, as well as Gram-negative bacteria, including *Escherichia coli*, *Salmonella paratyphi*, *Klebsiella pneumoniae*, and *Vibrio cholerae*, with inhibitory dosages varying depending on the specific PLA_2_ subtype [75,76].

In addition, Cathelicidins are a family of antimicrobial peptides that have been extensively studied in the venoms of snakes, particularly within the Elapidae and Viperidae families [78]. In 2008, Zhao and colleagues were the first to identify snake venom-derived cathelicidins. Their study revealed peptides isolated from both the venom and tissues of three Asian elapid species (Table 3). Key findings included NA-CATH from the Chinese cobra (*Naja atra*), OH-CATH from the king cobra (*Ophiophagus hannah*), and two peptides from the banded krait (*Bungarus fasciatus*), namely BF-CATH and cathelicidin-BF [40,79,80]. To date, 11 naturally occurring cathelicidins were identified and reported to possess significant antibacterial activity against Gram-positive and Gram-negative bacteria, including *S. aureus* and *E. coli* (Table 3) [78]. They are part of innate immunity and are characterized by a highly conserved anionic cathelin domain. These peptides exhibit a potent mechanism of action by rapidly compromising the integrity of microbial lipoprotein membranes. This is achieved through their ability to fuse with lysosomes within macrophages, leading to the swift destruction of the invading pathogens. Hc-cath, a cathelicidin peptide derived from the venom of the sea snake *Hydrophis cyanocinctus*, has demonstrated potent and broad-spectrum anti-microbial activity against a wide range of human pathogenic micro-organisms, with minimum inhibitory concentrations (MICs) ranging from 0.16 to 20.67 μM [38,39]. Remarkably, many of these pathogens resist to traditional anti-biotics, such as ampicillin. Hc-cath is composed of 30 amino acids, with the sequence KFFKRLLKSVRRAVKKFRKKPRLIGLSTLL. Hc-CATH has shown nearly equivalent efficacy against Gram-negative and Gram-positive bacteria [38]. Cathelicidin-BF, another potent cathelicidin-like anti-microbial peptide, is derived from the venom of the banded krait (*Bungarus fasciatus*) [40]. Its amino acid sequence, KFFRKLKKSVKKRAKEFFKKPRVIGVSIPF, is composed of 30 residues, featuring 12 basic residues (9 lysines and 2 arginines), 5 phenylalanines, and a single acidic residue (glutamic acid) [40]. This lysine and phenylalanine-rich composition contributes to its strong anti-microbial properties. Cathelicidin-BF has demonstrated potent antimicrobial activity, particularly against Gram-negative bacteria, including both standard strains and clinically isolated drug-resistant strains. Additionally, Cathelicidin-BF has shown significant antibacterial activity against Propionibacterium acnes, a common pathogen in skin infections [41]. OH-CATH30, a cathelicidin analog identified in the venom gland of the king cobra (*Ophiophagus hannah*), exhibits robust, salt-resistant antibacterial activity against both Gram-positive and Gram-negative bacteria, with MICs ranging from 1 to 20 µg/mL [80]. Additionally, OH-CATH30 demonstrates minimal hemolytic activity, with only about 10% hemolysis observed at a concentration of 200 µg/mL. Overall, these cathelicidins have shown potent anti-microbial activity both in vitro and in vivo, including effectiveness against several multidrug-resistant strains.

Crotamine, a highly basic peptide from the venom of *Crotalus durissus terrificus* rattlesnake, demonstrated low activity against both Gram-positive and Gram-negative bacteria, but showed potential as an anti-yeast or candicidal agent at low concentrations, with minimal harmful effects on normal mammalian cells [36]. Crotamine also exhibits potent anti-plasmodial activity, inhibiting the development of *Plasmodium falciparum* parasites in a dose-dependent manner, with an IC_50_ value of 1.87 μM [37]. The whole venom of *Crotalus durissus cumanensis*, along with fraction II containing crotoxin and Crotoxin B, exhibits anti-*Plasmodium falciparum* activity. The entire venom is effective at 0.17 ± 0.03 μg/mL, fraction II at 0.76 ± 0.17 μg/mL, and Crotoxin B at 0.6 ± 0.04 μg/mL [86].

**Figure 3:**
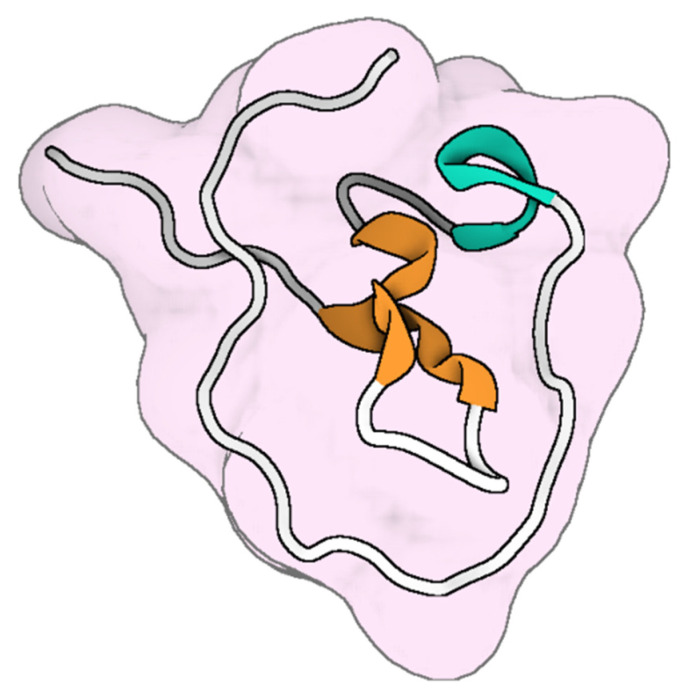
The molecular structure Omwaprin from *Oxyuranus microlepidotus* [87]. The colored parts reflect the secondary conformation structures of the peptides (the blue for the helices, orange for the β-sheets, hinges in cyan, and loops in gray). The molecular structure was visualized by BioRender. Al-Sabi, A. 2025. https://BioRender.com.

Omwaprin (Figure 3), a 50-amino-acid cationic protein from inland taipan (*Oxyuranus microlepidotus*) venom, exhibited selective, species-specific, and dose-dependent antibacterial activity against Gram-positive bacteria, with minimum inhibitory doses ranging from 2 to 10 μg in radial diffusion assays. It notably lacks hemolytic activity on human erythrocytes [43].

**Figure 4:**
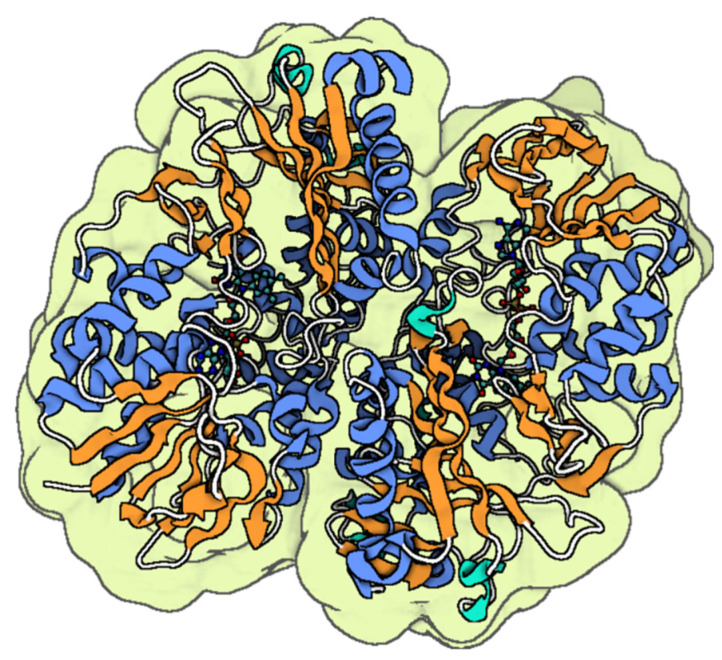
The molecular structure of L-amino acid oxidase (LAAO) from *Bothrops jararacussu* [89]. The colored parts reflect the secondary conformation structures of the peptides (the blue for the helices, orange for the β-sheets, hinges in cyan, and loops in gray). The molecular structure was visualized by BioRender. Al-Sabi, A. 2025. https://BioRender.com.

L-AAO isolated from *Bothrops marajoensis* venom (Figure 4) demonstrated inhibitory effects on the growth of *Pseudomonas aeruginosa*, *Candida albicans*, and *S. aureus*, as well as the parasitic growth of *Leishmania chagasi* and *Leishmania amazonensis* [15]. L-AAO from king cobra (*Ophiophagus hannah*) venom exhibited potent activity against Gram-positive bacteria, with MICs of 0.78 μg/mL (0.006 μM) and 1.56 μg/mL (0.012 μM) for *S. aureus* and *S. epidermidis*, respectively, but showed moderate efficacy against Gram-negative bacteria (*P. aeruginosa*, *K. pneumoniae*, and *E. coli*), with MICs ranging from 25 to 50 μg/mL (0.2–0.4 μM) [35].

Crotacetin, a novel C-type lectin homolog of convulxin from the venom of the South American rattlesnake *Crotalus durissus terrificus* and the first member of its family identified with antibacterial activity, inhibits the growth of the Gram-negative bacteria *Xanthomonas axonopodis pv. passiflorae* and *Clavibacter michiganensis* by 87.8% and 96.4%, respectively, at a concentration of 150 µg/mL [88].

Snake venom serine proteinases have not previously been associated with anti-microbial activity; however, a 27 kDa serine proteinase purified from the venom of the Peruvian snake *Bothriopsis oligolepis* showed an MIC of 80 µg/mL against *S. aureus* [90]. Venoms from *Bitis arietans*, *Naja haje*, and *Naja pallida* were tested against Gram-positive bacteria (*B. cereus, S. aureus*, and *Salmonella typhi*) and Gram-negative bacteria (*E. coli* and *Klebsiella pneumoniae*). *Naja haje* and *Naja pallida* venoms demonstrated superior antibacterial activity compared to some commonly used antibiotics, while *Bitis arietans* venom was ineffective against the tested pathogens [91]. These findings emphasize the potential of snake venom-derived components as promising antimicrobial agents, offering novel therapeutic prospects against resistant bacterial and fungal pathogens.

### 2.2. Antimicrobial Agents from Scorpions

Scorpion venom comprises several bioactive components, including peptides, nucleotides, amino acids, enzymes, lipids, mucoproteins, biogenic amines, and unknown substances [92]. Various scorpion species’ venoms have demonstrated antimicrobial effectiveness against both Gram-positive and Gram-negative bacteria, including *Enterococcus faecium*, *Streptococcus agalactiae*, *Micrococcus luteus*, *S. aureus*, *E. coli*, *Pseudomonas aeruginosa*, and *Enterobacter cloacae* [93].

Mucroporin-M1, a peptide derived from the venom of the Chinese swimming scorpion (*Lychas mucronatus*), possesses potent anti-microbial properties. Mucroporin-M1 is a 17-amino acid α-helical peptide (LFRLIKSLIKRLVSAFK) that was designed based on the protein sequence of mucroporin and exhibits amphipathic characteristics, allowing it to interact effectively with microbial membranes [45]. It has demonstrated broad-spectrum activity against Gram-positive and Gram-negative bacteria, including *S. aureus*, *E. coli*, *Pseudomonas aeruginosa*, and *Klebsiella pneumoniae* [45]. Beyond its antibacterial capabilities, it also exhibits antiviral effects, particularly against pathogens such as measles, severe acute respiratory syndrome coronavirus (SARS-CoV), hepatitis B virus, and influenza H5N1 viruses [46,47]. It demonstrated strong inhibitory effects, with EC50 values of 7.15 μg/mL (3.52 μM) for measles virus, 14.46 μg/mL (7.12 μM) for SARS-CoV, and 2.10 μg/mL (1.03 μM) for H5N1. Moreover, Mucroporin-M1 exhibited significantly enhanced anti-viral activity compared to the original mucroporin peptide, which lacked efficacy against these viruses. Three identified cysteine-free venom peptides (Pandinin 1, Pandinin 2, and Pandinin 3) from the venom of the African scorpion *Pandinus imperator* exhibit potent bactericidal effects against Gram-positive bacteria. They are also effective against the fungus *Candida albicans*. However, these peptides demonstrate minimal antimicrobial activity against Gram-negative bacteria [48].

BmKn2 is an anti-microbial peptide (AMP) derived from the venom of the Manchurian scorpion (*Mesobuthus martensii* Karsch) [49]. It has vigorous antibacterial activity against *S. aureus*, *Micrococcus luteus*, *B. subtilis, E. coli*, *Pseudomonas aeruginosa*, and *Neisseria gonorrhoeae*. To enhance its antibacterial potency and minimize hemolytic activity, a derivative called BmKn2-7 was developed. BmKn2-7 demonstrated significantly improved inhibitory activity against Gram-positive and Gram-negative bacteria compared to the original BmKn2 [50]. Additionally, BmKn2-7, but not BmKn2, exhibited strong inhibition of HIV-1 through direct interaction with viral particles [51].

AaeAP1 and AaeAP2, isolated from the North African scorpion *Androctonus aeneas*, contain 17 amino acids and lack disulfide bridges. They show more selective growth-inhibitory activities against the Gram-positive bacterium *S. aureus* (16 mg/L) and the yeast *Candida albicans* (32 mg/L) compared to the Gram-negative bacterium *E. coli* (512 mg/L) [52].

Opistoporin 1 and parabutoporin, pore-forming peptides isolated from the venom of the South African scorpion *Parabuthus schlechteri*, exhibited activity in inhibiting the growth of Gram-negative bacteria (1.3–25 µM) [94]. Imcroporin, a cationic anti-microbial peptide derived from the venom of the scorpion *Isometrus maculatus*, has proven potent growth-inhibitory activity against antibiotic-resistant bacteria, particularly methicillin-resistant *S. aureus*, while showing minimal effect on Gram-negative bacteria and relatively low hemolytic activity against human erythrocytes [53].

Opisin, an antimicrobial peptide isolated from the scorpion *Opistophthalmus glabrifrons*, comprises 19 amino acid residues and lacks disulfide bridges. It inhibits the growth of tested Gram-positive bacteria, with MIC values ranging from 4.0 to 10.0 μM. In contrast, it exhibits significantly lower activity against the tested Gram-negative bacteria and fungi [95]. Additionally, over 20 peptides derived from scorpions have been shown to possess significant antifungal activity, while 10 peptides with antiparasitic properties have been reported to date [96,97]. Scorpion venom thus represents a promising reservoir of antimicrobial compounds with potential applications in pharmaceutical research and development.

Gao et al. [98] isolated two linear cationic peptides, meucin-24 and meucin-25, from the venom glands of *Mesobuthus eupeus*, which have antimalarial activity and low cytotoxicity. These peptides, similar to frog skin magainins, inhibited *P. berghei* development and killed *P. falciparum* at micromolar concentrations without affecting human GC-2 cell viability at 50 μM or causing hemolysis in mouse erythrocytes at 100 μM.

### 2.3. Antimicrobial Agents from Spiders

Recent studies have shed light on the potential of anti-microbial peptides found in spider venoms as a valuable resource for developing novel anti-infective compounds capable of combating drug-resistant microbes [99,100]. Spider venom serves as a promising reservoir of antimicrobial agents. Numerous anti-microbial peptides have been identified in various spider species, including *Lachesana tarabaevi*, *Cupiennius salei*, *Oxyopes takobius*, *Hogna carolinensis*, *Lycosa erythrognatha*, and *Lycosa singoriensis* [100].

Several lycotoxins have been identified for their potent anti-microbial properties. Particularly, M-lycotoxin-Ls3a, derived from the venom of *Lycosa singoriensis*, and inhibits the growth of both Gram-positive (*S. aureus* and *B. subtilis*) and Gram-negative (*E. coli* and *Pseudomonas aeruginosa*) bacteria, as well as fungi such as *Candida albicans*, at micromolar concentrations [54]. The MICs of M-lycotoxin-Ls3a against *E. coli*, *Pseudomonas aeruginosa*, and *B. subtilis* range from 3.14 to 6.29 µM, 5.02–10.05 µM, and 1.57–3.14 µM, respectively, while for *Candida albicans*, the MIC is between 1.25 and 2.51 µM [54]. Another lycotoxin, M-lycotoxin-Hc1a, a 25-amino acid peptide from the Carolina wolf spider (*Hogna carolinensis*), also exhibits anti-microbial activity [55]. These lycotoxins share an amphipathic alpha-helical structure, characteristic of antimicrobial pore-forming peptides, contributing to their ability to disrupt microbial membranes.

Lytx-Pa2a, a peptide identified from the venom of the spider *Pardosa astrigera*, demonstrated potent antibacterial activity against both Gram-negative (*E. coli* and *Pseudomonas aeruginosa*) and Gram-positive bacteria (*B. cereus* and *S. aureus*) [101]. U1-SCRTX-Lg1a is a peptide isolated from the venom of the spider *Loxosceles gaucho*, exhibiting selective antimicrobial activity against Gram-negative bacteria, including *E. coli*, *Pseudomonas aeruginosa*, and *Enterobacter cloacae*. It shows no hemolytic activity against human erythrocytes or cytotoxicity toward HeLa cells. The peptide is active within a concentration range of 1.15 μM (1.9 μg/mL) to 4.6 μM (7.6 μg/mL), with *Pseudomonas aeruginosa* being the most sensitive, displaying a MIC of 1.15 μM (1.9 μg/mL). However, U1-SCRTX-Lg1a is ineffective against Gram-positive bacteria such as *Micrococcus luteus*, *S. aureus*, and *B. subtilis*, as well as fungi (*Aspergillus niger*) and yeasts (*Candida albicans* and *Candida krusei*) [56].

AATX-Ab2a and AATX-Ab3a were identified from the venom gland transcripts of the *Argiope bruennichi* spider through an in silico approach. Experimental validation confirmed that both peptides exhibit strong inhibitory effects against a broad spectrum of pathogens, including Gram-positive bacteria (*B. cereus* and *S. aureus*), Gram-negative bacteria (*E. coli* and *Pseudomonas aeruginosa*), multidrug-resistant *P. aeruginosa*, and fungal species such as *Candida albicans* and *Fusarium oxysporum* [102]. This highlights the rich potential of spider venom as a source of diverse antimicrobial compounds with broad applications in combating infectious diseases.

### 2.4. Anti-Microbial Agents from Honeybees

**Figure 5:**
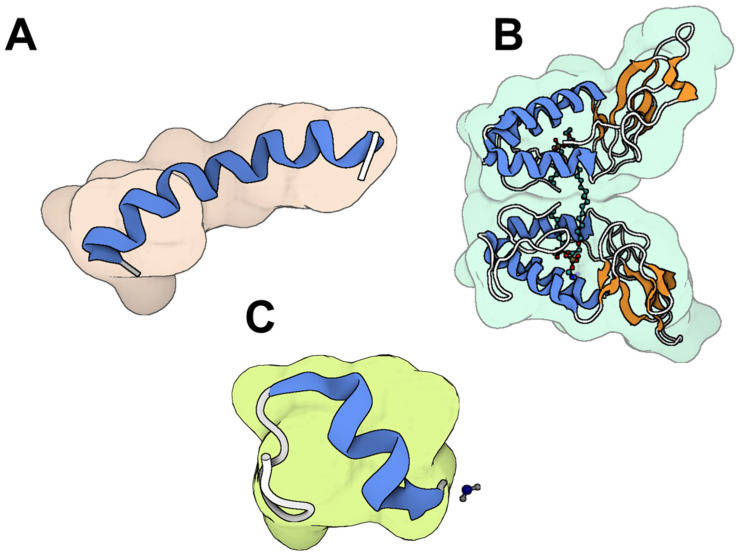
The molecular structure of representative honey bee peptides (**A**) melittin [108], (**B**) PLA_2_ [109] and (**C**) apamin [110] from *Apis mellifera*. The colored parts reflect the secondary conformation structures of the peptides (the Blue for the helices, orange for the β-sheets, hinges in cyan, and loops in gray). The molecular structure was visualized by BioRender. Al-Sabi, A. 2025. https://BioRender.com.

Honeybee venom, a complex biotoxin, comprises a diverse range of dynamic components, including proteins (PLA_2_ and hyaluronidase), small peptides (such as apamin and melittin) (Figure 5), as well as amines (histamine, dopamine, norepinephrine), and amino acids [103,104]. Bee venom (BV) has been used therapeutically since Ancient Egypt. Its applications were further expanded by notable figures (Hippocrates, Aristotle, and Galen) during Greek and Roman times, as well as in Traditional Chinese Medicine and other ancient practices, particularly for treating inflammatory conditions such as rheumatoid arthritis, tendonitis, fibrosis, lupus, and multiple sclerosis [105]. BV exhibits remarkable anti-microbial effects primarily attributed to melittin, a key peptide component [57,58]. Melittin enhances cell permeability by integrating into phospholipid bilayers at low concentrations, ultimately creating pores in cell membranes [58]. BV exhibits potent antibacterial effects against Methicillin-resistant *S. aureus* (MRSA) strains CCARM 3366 and CCARM 3708. The MIC of BV was determined to be 0.085 µg/mL for MRSA CCARM 3366 and 0.11 µg/mL for MRSA CCARM 3708, while the minimum bactericidal concentrations (MBC) were 0.106 µg/mL and 0.14 µg/mL for the respective strains [106]. The antibacterial properties of BV and purified melittin against *E. coli* and *S. aureus* were evaluated by determining their MICs and post-antibiotic effects (PAEs). The PAEs of whole BV against *E. coli* were 0.15 h at 1 × MIC and 2.4 h at 5 × MIC, while for *S. aureus*, it was 3.45 h at 1 × MIC. Melittin showed PAEs of 0.1 h at 1 × MIC and 3.2 h at 5 × MIC for *E. coli*, and 4.35 h at 1 × MIC for *S. aureus* [107].

BV and its primary components, melittin and PLA_2_, were evaluated against oral bacteria linked to tooth decay, with BV showing MICs ranging from 20 to 40 µg/mL for pathogens such as Streptococcus species (*S. salivarius*, *S. sobrinus*, *S. mutans*, *S. mitis*, and *S. sanguinis*), *Lactobacillus casei*, and *Enterococcus faecalis*. Melittin demonstrated MIC values between 4 and 40 µg/mL, while PLA_2_ required concentrations above 400 µg/mL. Additionally, both BV and melittin altered the structure and size of *Borrelia burgdorferi* biofilms, the bacteria behind Lyme disease, while anti-biotics often led to relapse after treatment was stopped [63,111]. BV’s constituents exhibit robust antiviral properties, effectively targeting a broad spectrum of viruses. BV and its main components demonstrate significant anti-viral properties against various enveloped and non-enveloped viruses, including influenza A virus and Respiratory Syncytial Virus, as well as Vesicular Stomatitis virus, Herpes Simplex virus, Enterovirus-71, and Coxsackievirus [112]. Moreover, BV has emerged as a potent therapeutic agent for combating fungal infections, demonstrating efficacy against various fungal illnesses. BV effectively inhibits dermatophytosis caused by Trichophyton mentagrophytes and Trichophyton rubrum, demonstrating greater potency than fluconazole, a commonly used antifungal medication [113]. BV exhibited MIC against ten clinical isolates of Candida albicans ranging from 62.5 to 125 μg/mL [114]. Additionally, melittin displayed antimicrobial activity against various fungal strains, with MIC values between 30 and 300 μg/mL [115]. Also, BV and melittin present promising therapeutic options for treating microbial infections. However, their potential clinical application is hindered by significant hemolysis and cellular toxicity, which pose grave side effects that need to be addressed for their future development.

### 2.5. Antimicrobial Agents from Wasps

Wasps belong to the Hymenoptera, the third-largest order of insects. They are equipped with venom containing a diverse array of bioactive compounds [116]. This venom comprises proteins, enzymes, small peptides, and low molecular weight molecules, including bioactive amines, amino acids, and various potent antibacterial agents [116]. Remarkably, venom contains anti-microbial peptides, which exhibit broad-spectrum activity against both Gram-negative and Gram-positive bacteria, as well as viruses, fungi, and protozoa [117,118]. The venom of wasps (*Vespa orientalis*) demonstrated vigorous antimicrobial activity, with Gram-positive bacteria (*B. cereus* and *S. aureus*) being more sensitive than Gram-negative bacteria (*Salmonella typhimurium* and *E. coli*) and the fungal strain (*Candida albicans*). The highest inhibition zones were 29.3 mm for B. cereus and 24.3 mm for *S. aureus*. Minimum inhibitory concentrations ranged from 0.16 to 1.25 mg/mL, with *B. cereus* having the lowest value at 0.16 mg/mL [65]. Another study on *Vespa orientalis* venom also highlighted its antimicrobial properties. In this case, Gram-positive bacteria, such as *S. aureus* and *B. subtilis*, were more sensitive than Gram-negative strains (*E. coli* and *Klebsiella pneumonia*). The inhibition zones reached 22.7 mm for *B. subtilis* and 12.6 mm for *S. aureus*, with MIC as low as 8 µg/mL for *B. subtilis* [119].

Forty mastoparan sequences have been identified in the venoms of social wasps, while six have been found in solitary wasps. Mastoparan-L, derived from the venom of *Vespula lewisii*, exhibits more potent toxicity in *S. aureus* than in *E. coli* and human erythrocytes by promoting K^+^ efflux and phospholipid release [120]. Another example is the Mastoparan-M peptide, which exhibited broad-spectrum antimicrobial activity against Gram-positive and Gram-negative bacteria [121]. Mastoparan B (MP-B), an amphiphilic alpha-helical peptide isolated from the hornet *Vespa basalis*, demonstrated significant anti-microbial efficacy against both Gram-positive and Gram-negative bacteria, with MIC values of 3.3 mg/mL against *Enterococcus faecalis* LS-101 and *B. subtilis* PCI 219, and 6.25 mg/mL against *Shigella flexneri* EW-10 and *Shigella sonnei* EW-33 [66]. Mastoparan-VT1, isolated from *Vespa tropica* and closely related to mastoparan-M from *Vespa mandarinia*, demonstrated extensive anti-microbial activity against various standard and clinically isolated strains. The scored MIC values varied between 2.5 and 10 μg/mL for Gram-positive bacteria, 5 to 40 μg/mL for Gram-negative bacteria, and 10 to 40 μg/mL for different Candida strains [122].

Two natural AMPs derived from the venom of the solitary eumenine wasp *Eumenes micado* were successfully converted into α-helical AMPs with reduced toxicity, effectively targeting and killing the Gram-negative pathogens *E. coli* and *Pseudomonas aeruginosa* [123]. Polybia-CP, a 12-amino acid peptide from the venom of the wasp *Polybia paulista*, exhibits antimicrobial and chemotactic properties. It effectively combats Gram-positive bacteria, such as *S. aureus* and *B. subtilis*, as well as Gram-negative bacteria, such as *E. coli* and *Pseudomonas aeruginosa* and *S. epidermidis* [67,68]. However, it demonstrates significant toxicity to mammalian cells. Researchers have used a physicochemical-guided design strategy to address this issue by reducing its toxicity while maintaining its antibacterial efficacy [124]. This emphasizes the therapeutic potential of wasp venom-derived compounds in combating infectious diseases.

### 2.6. Antimicrobial Agents from Ants

Ant venom comprises a diverse array of bioactive compounds, including proteins, peptides, hydrocarbons, free amino acids, biogenic amines, formic acid, salts, and sugars [125]. However, stinging ants of the genera Solenopsis and Monomorium stand out for producing venom with fewer proteins and a higher alkaloid content compared to other ant species [126,127]. Peptides derived from ant venom exhibit notable antimicrobial properties [71,128]. The crude venom of *Pachycondyla goeldii* demonstrated intense antimicrobial activity, with the most sensitive Gram-positive bacteria being *Geobacillus stearothermophilus*, *B. subtilis*, *B. megaterium*, and *Lactococcus lactis*, while *Pseudomonas aeruginosa* was the most sensitive Gram-negative bacterium [71]. Fifteen novel peptides, named ponericins, exhibiting antibacterial and insecticidal properties, were purified from the venom of the predatory ant *Pachycondyla goeldii*. According to their primary structure similarities, they can be classified into three families: ponericin G, W, and L [71].

Bicarinalin is an antimicrobial peptide isolated from the venom of the ant *Tetramorium bicarinatum* and, structurally, it is a linear, cysteine-free peptide composed of 20 amino acid residues. This peptide demonstrates a broad spectrum of activity against various micro-organisms, including bacteria, fungi, and parasites, with MICs ranging from 2 to 25 μmol/L. Bicarinalin effectively targets multiple bacterial strains, such as *Staphylococcus*, *Enterobacteriaceae*, and *Cronobacter sakazakii*, as well as resistant strains of *Pseudomonas aeruginosa*. Additionally, it exhibits antifungal activity against *Candida albicans* and *Aspergillus niger*, and it is active against the parasite *Leishmania infantum*, with a minimal inhibitory concentration of 2 μmol/L [72,73]. Remarkably, Bicarinalin has low hemolytic activity against human red blood cells. Furthermore, bicarinalin shows promise as an anti-infective agent against antibiotic-resistant pathogens. For instance, research indicates it is effective against *Helicobacter pylori*, the bacterium responsible for various gastric diseases. Also, Bicarinalin inhibits the adherence of *H. pylori* to gastric cells while exhibiting low toxicity to human cells [129].

Solenopsins are a group of alkaloids found in the venom of fire ants (*Solenopsis invicta*). These compounds can inhibit the growth of various bacteria, including *Streptococcus pneumoniae*, *S. aureus*, *Enterococcus faecalis*, and *Stenotrophomonas maltophilia* [69]. Additionally, natural and synthetic solenopsins have shown effectiveness against *Candida auris* strains from different clades, including those resistant to fluconazole and amphotericin B. Moreover, these alkaloids inhibit matrix deposition and alter the cellular metabolic activity of *C. auris* under biofilm conditions [70]. This emphasizes the complex mechanisms ant employs in combating microbial threats, highlighting their potential in antimicrobial research and drug development.

## 3. Mechanisms of Action of Venom-Based Anti-Microbial Agents

**Figure 6:**
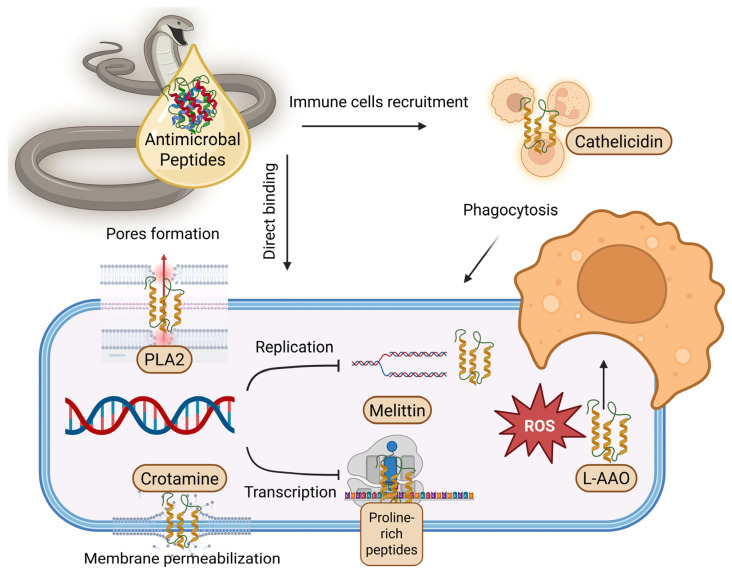
Mechanisms of action of animal venom-derived antimicrobial peptides. This schematic highlights the diverse mechanisms by which animal venom-derived antimicrobial peptides contribute to pathogen neutralization and immune modulation. Key actions include pore formation (e.g., PLA_2_), membrane permeabilization (e.g., crotamine), inhibition of DNA replication and transcription (e.g., melittin and proline-rich peptides), generation of reactive oxygen species (ROS) (e.g., L-amino acid oxidase), and promotion of phagocytosis (e.g., cathelicidins). Created in BioRender. Hegazy, A.M. 2025. https://BioRender.com.

Venom, usually linked to danger and toxicity, contains bioactive compounds with remarkable biological properties [14,118]. These venom-derived molecules have evolved over millions of years to serve specific functions, including prey capture and defense. AMPs hold immense promises for combating MDR pathogens due to their unique and multifaceted mechanisms of action [130,131]. Unlike traditional antibiotics that often target a single mechanism of action in microbes, venom-derived AMPs employ a comprehensive arsenal to take MDR pathogens (Figure 6). Their primary weapon is membrane disruption [132]. This disrupts the membrane’s integrity, leading to the leakage of vital cellular contents and, ultimately, cell death.

Furthermore, some venom-derived AMPs can act as molecular disruptors, inhibiting the synthesis or integrity of the bacterial cell wall [133]. This critical structure, essential for maintaining shape and protection, becomes compromised, leaving the bacteria vulnerable to external factors. Venom-derived AMPs can also target the core of microbial existence—essential intracellular processes [134]. They might disrupt protein synthesis by targeting ribosomes, inhibit DNA replication, or interfere with crucial signaling pathways [135]. By disrupting these processes, venom-derived AMPs effectively impede the microbe’s ability to function and replicate.

The power of venom-derived AMPs goes beyond direct microbial attack. Some venom-derived AMPs exhibit immunomodulatory properties, meaning they can work in tandem with the host’s immune system [136]. They might recruit immune cells to the infection site, enhance the engulfment of microbes (phagocytosis), or directly activate immune defenses.

### 3.1. Antibacterial and Antifungal Mechanisms of the Action of Animal Venom

**Figure 7:**
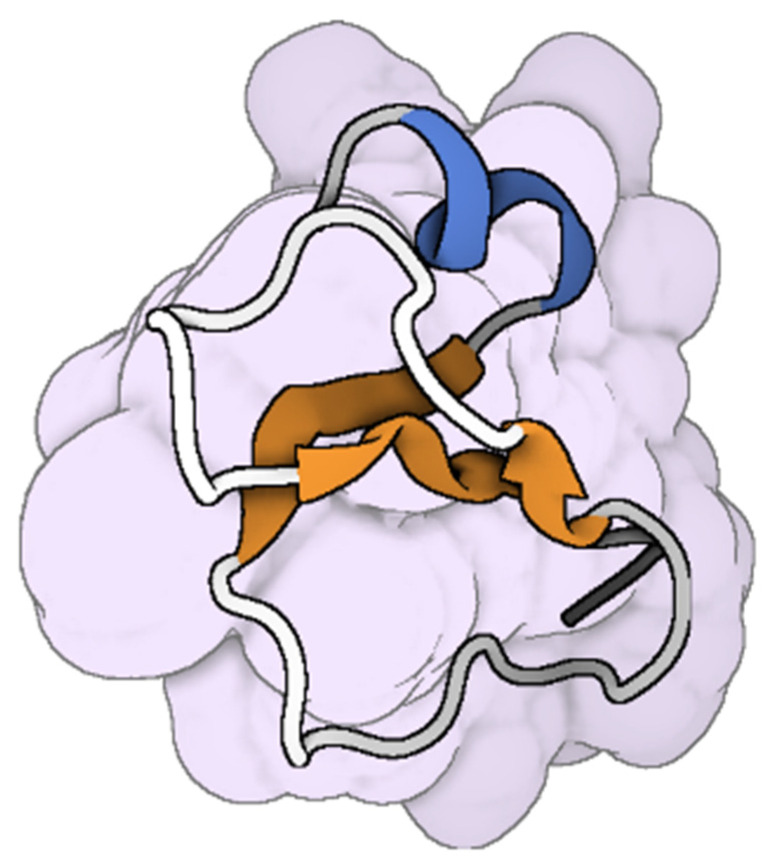
The molecular structure of crotamine from *Crotalus durissus terrificus* [143]. The colored parts reflect the secondary conformation structures of the peptides (blue for the helices, orange for the β-sheets, and loops in gray). The molecular structure was visualized by BioRender. Al-Sabi, A. 2025. https://BioRender.com.

Snake venom contains effective enzymes such as PLA_2_s, which can form pores in microbial cell membranes, leading to cell apoptosis [76,137]. The *Crotalus adamanteus* toxin-II (CaTx-II), a basic PLA_2_ enzyme from *Crotalus adamanteus* venom, effectively disrupts bacterial cell walls of *S. aureus*, *Burkholderia pseudomallei*, and *Enterobacter aerogenes*, while exhibiting low toxicity to normal lung, skin fibroblast cells [44]. Moreover, crotamine, a myotoxin from the venom of South American rattlesnake (*Crotalus durissus terrificus*; Figure 7), inhibits the growth of *E. coli* through membrane permeabilization, with a MIC of 25-100 mg/mL and no hemolytic effects [138]. Short peptides derived from the C-terminal region of *Bothrops asper* myotoxin II, specifically Lys49 and Asp49 PLA_2_, have been shown to interact functionally with bacterial lipo-polysaccharide (LPS). Initial sequence modification, introducing a triple Tyr to Trp substitution, significantly increased the peptides’ bactericidal potency [139,140]. King cobra venom L-AAOs exert their antibacterial effects by generating hydrogen peroxide through their oxidative activity, inducing oxidative stress in target cells [35]. Omwaprin disrupts bacterial membranes, a mechanism of action confirmed through scanning electron microscopy [43]. Crotamine, with a positive net charge on its surface, binds to negatively charged membranes, causing perturbation, disruption, and eventual rupture of the target membrane, without involving membrane permeabilization, and demonstrates activity against *Candida* spp. [141]. Snake venom-derived cathelicidins, such as NA-CATH, OH-CATH, Hc-CATH, SA-CATH, CATHPb1, and cathelicidin-BF, demonstrate potent antimicrobial activity through a dual mechanism involving direct pathogen targeting and modulation of host immune responses [79]. Mechanistically, these peptides initially exert their effects by interacting with negatively charged microbial membranes, causing membrane destabilization, pore formation, and subsequent cell lysis. Beyond their direct antimicrobial actions, snake cathelicidins critically modulate innate immune pathways [142]. For example, the sea snake cathelicidin Hc-CATH not only inhibits microbial growth, but also suppresses the production of pro-inflammatory cytokines such as tumor necrosis factor α (TNF-α), interleukin-1 (IL-1), interleukin-6 (IL-6), and nitric oxide (NO) following lipopolysaccharide (LPS) stimulation [38]. Hc-CATH directly binds to LPS, Toll-like receptor 4 (TLR4), and myeloid differentiation factor 2 (MD2), thereby inhibiting LPS-induced inflammatory signaling through the TLR4/MD2 complex. Similarly, SA-CATH significantly reduces LPS-induced production of pro-inflammatory cytokines in mouse peritoneal macrophages, while CATHPb1 enhances immune defense by recruiting macrophages and neutrophils to infection sites, promoting their proliferation and enhancing bactericidal activity, often synergizing with cytokines or β-defensins [82,83].

Hadrurin, a peptide from the venom of the scorpion *Hadrurus aztecus*, exhibits antimicrobial properties through a membrane destabilization mechanism. It inhibits the growth of bacteria, including *Salmonella typhi*, *Klebsiella pneumoniae*, *Enterococcus cloacae*, *Pseudomonas aeruginosa*, *E. coli*, and *Serratia marcescens* [144]. Androctonin, a peptide extracted from the hemolymph of *Androctonus australis* scorpions, has demonstrated activity against both bacteria (Gram-positive and Gram-negative) and fungi. Initially, the peptide interacts electrostatically with the target membrane, losing its β-sheet structure. It then aligns parallel to the lipid monolayer, causing membrane permeabilization and the subsequent efflux of potassium ions [145].

The wolf spider (*Lycosa carolinensis*) produces antimicrobial peptides in its venom, known as lycotoxins-I and II. These lycotoxins form pores in microbial cell membranes, causing calcium ion efflux and dissipating voltage gradients by altering membrane permeability. These mechanisms exhibit anti-microbial activity against bacteria, such as *E. coli*, and yeast (*Candida glabrata*) [146].

Melittin is a linear cationic peptide consisting of 26 amino acid residues. Identified around 1970, it is a significant component of the venom of the European honeybee, Apis mellifera [147]. Melittin is one of the most thoroughly researched AMPs [148]. It has shown broad-spectrum bactericidal activity, effective against reference and clinical bacterial strains, including antibiotic-resistant bacteria such as *Acinetobacter baumannii* and *Pseudomonas aeruginosa* [149]. Consequently, melittin is commonly used as a positive control in assessing the anti-microbial activity of newly discovered or developed AMPs. Melittin causes cell lysis through a pore-formation mechanism in various bacterial and fungal strains [150]. Melittin targets phosphatidylcholine in bacterial cell membranes due to the positive charge of its N-terminus, supporting attachment and subsequent pore formation or downregulating protein phosphorylation, resulting in cell disruption [19,20]. Melittin also inhibits methicillin-resistant *S. aureus* [151]. Additionally, melittin penetrates compromised membranes to interfere with DNA replication by binding DNA, inhibiting polymerase and topoisomerase activity, and potentially disrupting transcription [152].

Although ant venom can be extracted only in small quantities due to the ants’ size, it has significant pharmacological uses. It has been used as a Chinese anti-inflammatory medicine for a long time. Ponericins are a set of 15 peptides identified in the venom of the predatory ant *Pachycondyla goeldii*, categorized into three families based on similarities in their primary structures: ponericins G, W, and L. Ponericins G share significant similarities with cecropins, while ponericins L are like dermaseptins. Both groups exhibit potent antibacterial activity, using a “carpet-like” mechanism to disrupt cell membranes [71]. P17, a peptide derived from the venom of the ant *Tetramorium bicarinatum*, activates the arachidonic acid (AA)/leukotriene B4 (LTB4)/peroxisome proliferator-activated receptor gamma (PPARγ)/Dectin-1-mannose receptor (MR) axis. This activation induces the production of reactive oxygen species (ROS) and inflammasome-dependent interleukin (IL)-1β, enhancing the ability to recognize and engulf the fungus *Candida albicans*, resulting in fungal cell death [153]. Bicarinalin, another peptide from the same ant venom, exhibited broad-spectrum antibacterial and antifungal activities, and antiparasitic effects against *Leishmania infantum* by permeabilizing cell membranes [153].

### 3.2. Antiviral Mechanisms of the Action of Animal Venom

Components of snake venom, such as L-AAO, PLA_2_, and metalloproteases, demonstrate anti-viral properties against HIV and Dengue virus. PLA_2_s from *Bothrops leucurus* snake venom have been shown to reduce the amount of DENV RNA in infected cells [154]. Venom sPLA_2_s, such as NmmCMIII from the *Naja mossambica mossambica* and taipoxin from the *Oxyuranus scutellatus*, have significant inhibitory effects on HIV-1 infection. These sPLA_2_s can protect various host cell types from the replication of primary HIV-1 isolates [155]. Similarly, PLA_2_ from the honeybee *Apis mellifera* has also been shown to prevent the intracellular release of the viral capsid proteins of both HIV and hepatitis C virus (HCV) [155]. The melittin peptide from bee venom can inhibit viral replication in both enveloped and non-enveloped viruses in vitro, and it has also been shown to limit the influenza A virus H1N1 [112]. A new antiviral peptide Smp76, derived from the venom of the Egyptian scorpion *Maurus palmatus*, exhibits anti-viral properties against viruses in the Flaviviridae family, including HCV and dengue virus (DENV). Smp76 can inhibit DENV infection before viral entry, making it a potential treatment for DENV viremia [156]. The *Alopecosa nagpag* spider produces a defense peptide named Antiviral-Lycotoxin-An1a (Av-LCTX-An1a), which inhibits the protease NS2B-NS3 of DENV and Zika virus, preventing flavivirus infection [157].

### 3.3. Antiparasitic Mechanisms of the Action of Animal Venom

Mastoparan from *Polybia paulista* wasp venom functions as both an antibacterial and antiparasitic agent by inhibiting the formation of the vital enzyme glyceraldehyde-3-phosphate dehydrogenase in *Trypanosoma cruzi*. Additionally, melittin has been reported to eradicate parasites through its immunomodulatory effect on macrophages. On the other hand, ant venom contains the bicarinalin peptide, which exhibits antibacterial and antifungal properties via membrane permeabilization. It also shows antiparasitic activity against *Leishmania infantum*. The venom of the *Crotalus durissus terrificus* snake contains a cathelicidin peptide named crotalicidin, which exerts antiparasitic effects against *Trypanosoma cruzi* through necrosis, marked by membrane disruption and loss of integrity. Furthermore, *Crotalus durissus terrificus* snake venom includes the crotamine peptide, known for its antiparasitic activity, particularly antiplasmodial activity. Crotamine is localized in the nucleus and porous vacuoles of the parasite, with its antimalarial mechanism of action thought to induce apoptosis through disruption of the parasite’s acidic compartment H^+^ homeostasis [37].

## 4. Synergistic Interactions and Combination Therapies

Animal venoms represent a promising frontier in the fight against antimicrobial resistance due to their unique evolutionary adaptations and diverse mechanisms of action (Table 4). Venom-derived peptides, such as melittin from honeybees and cathelicidins from snake venoms, exhibit broad-spectrum activity against bacteria, fungi, and even drug-resistant pathogens like Methicillin-resistant *S. aureus* and *Candida albicans*. Unlike conventional antibiotics, which often target specific molecular pathways prone to resistance, venom components disrupt microbial membranes through pore formation or enzymatic degradation, making it more difficult for pathogens to develop resistance. Additionally, some venom peptides modulate host immune responses, enhancing the body’s natural defenses while suppressing biofilm formation. The ability of venom peptides to act synergistically with existing antibiotics further amplifies their therapeutic potential. Despite challenges such as toxicity and stability, advancements in peptide engineering and delivery systems are unlocking safer, more effective venom-based therapies.

Combining venom-derived peptides with commercial antibiotics has emerged as an effective strategy to combat resistant bacteria. This approach enhances the activity of existing antibiotics and provides a multifaceted solution by targeting resistance mechanisms. Combination therapy offers several advantages, including increased efficacy, rapid clinical application, lower doses, excellent stability, and fewer side effects compared to conventional antibiotics. By neutralizing bacterial resistance mechanisms while enhancing the efficacy of antibiotics, this synergistic approach provides more effective treatments for life-threatening infectious diseases.

### 4.1. Css54 AMP

Css54 AMP was isolated and purified from the crude venom of the scorpion *Centruroides suffusus*, and then combined with commercial antibiotics (e.g., Rifampin, Isoniazid, and Pyrazinamide) [163]. Css54 exhibited the best results when paired with the antibiotic rifampicin. The treatment of *S. aureus* with Css54 (14 mg/mL) combined with rifampicin alone demonstrated a synergistic effect. In contrast, the combining Css54 peptide (14 mg/mL) with rifampicin, isoniazid, pyrazinamide, and ethambutol showed only an additive effect. Although Mycobacterium tuberculosis shows strong resistance to rifampicin, the combination of Css54 AMP and rifampicin proved effective in treating tuberculosis.

### 4.2. Macropin

Macropin, isolated from the venom of the solitary bee *Macropis fulvipes* (Hymenoptera: Melittidae), demonstrated improved inhibition of bacterial growth when combined with commercial antibiotics (e.g., Gentamycin, Tobramycin, Ciprofloxacin, Levofloxacin, Piperacillin, or Oxacillin) [164]. Combining Macropin with oxacillin showed a partial synergistic effect with a 0.52 fractional inhibitory concentration index against *S. aureus*. However, combinations with other antibiotics such as gentamicin, tobramycin, ciprofloxacin, levofloxacin, piperacillin, or oxacillin resulted in an additive effect against *S. aureus* bacteria. Treatment of *S. aureus* and *Pseudomonas aeruginosa* with macropin-induced membrane blebs, damage, atrophy, destruction, and shrinking, suggesting the peptide’s ability to affect bacterial membrane integrity [164]. Combination therapy with Macropin and antibiotics demonstrated antibacterial potential at lower doses compared to when the peptide or antibiotics were used alone. Overall, the antibiotics and Macropin exhibited partial synergistic or additive effects.

### 4.3. Honeybee Venom and Its Melittin

The remarkable efficacy of honeybee venom and its constituents has prompted scientists to explore potent evolutionary combinations for treating multidrug-resistant bacterial infections. Synergistic effects are observed when honeybee venom is combined with antibiotics or selected plant secondary metabolites, chosen for their diverse mechanisms of action. Compounds such as sanguinarine and berberine, extracted from *Chelidonium majus* L. and *Berberis thunbergii* DC, exhibit growth inhibition of microbes by damaging their DNA, with fractional inhibitory concentration indexes ranging from 0.24 to 0.5 [165]. However, combinations of honeybee venom with berberine against methicillin-resistant *S. aureus* or with amikacin against rapidly growing mycobacteria show additive properties, with combination indexes ranging from 0.75 to 1 [115]. In another instance, adding half the minimum inhibitory concentration of honeybee venom to half the minimum inhibitory concentration of vancomycin demonstrates a synergistic effect against vancomycin-resistant enterococci after 24 h [166]. The most effective triple drug combination involves treating vancomycin-resistant enterococci with half the minimum inhibitory concentration of vancomycin, combined with a quarter of the minimum inhibitory concentration of honeybee venom and a quarter of the minimum inhibitory concentration of benzyl isothiocyanate (a plant secondary metabolite), resulting in a 104 decrease in colony counts after 24 h [115]. Studies have shown that honeybee venom, when combined with kanamycin and ampicillin, acts synergistically against kanamycin-resistant *S. aureus* and *E. coli*, respectively, and prolongs the half-life of antibiotics [167].

Melittin was combined with either carvacrol or benzyl isothiocyanate against methicillin-resistant *S. aureus* and *E. coli* bacteria, resulting in fractional inhibitory concentration index values ranging between 0.24 and 0.5. Furthermore, research found that melittin exhibits synergistic effects when paired with amoxicillin and cefuroxime against Gram-positive bacteria and, similarly, when combined with erythromycin against Gram-negative bacteria [168]. Melittin also demonstrates notable synergism when administered with lactam antibiotics or polymyxin B against multidrug-resistant bacteria.

Despite increasing interest in venoms for their antibacterial potential, no therapeutic approach has yet progressed to the clinical trial stage. However, the diverse mechanisms of action exhibited by these therapeutic combinations offer promising avenues for overcoming multidrug-resistant microbial challenges and restoring antibiotics efficacy.

## 5. Future Prospects and Challenges of Venom-Derived AMPs

Venom-derived AMPs represent a promising frontier in the fight against multidrug-resistant pathogens due to their unique mechanisms of action, rapid bactericidal activity, and reduced potential for resistance development. However, despite their therapeutic potential, translating these peptides into clinical applications remains fraught with several key challenges.

### 5.1. Pharmacokinetic Barriers and Administration Challenges

A major limitation of peptide-based therapeutics is their poor oral bioavailability, primarily due to enzymatic degradation in the gastrointestinal tract and limited permeability across epithelial barriers. Innovative delivery strategies, such as encapsulation in nanoparticles or conjugation with cell-penetrating peptides, are being explored to overcome these barriers. For example, coupling melittin with nanoparticles has enhanced its in vivo stability and reduced off-target effects, opening doors for broader therapeutic applications [169]. Moreover, co-formulating AMPs with immunomodulatory or synergistic agents may enhance both efficacy and delivery potential.

### 5.2. Toxicity and Selectivity Issues

One of the major limitations in the clinical applications of venom-derived AMPs is their inherent cytotoxicity and lack of selectivity, which can result in damage to host cells, including hemolysis and tissue injury. Melittin, for instance, while effective against a wide range of pathogens, exhibits strong hemolytic activity that restricts its systemic use. To address this, several innovative strategies have been employed to improve selectivity and reduce toxicity. Peptide engineering approaches, including amino acid modifications to optimize charge and hydrophobicity, and the design of truncated analogs, have shown promise in preferentially targeting bacterial membranes over mammalian cells [170]. Modifications such as N- and C-terminal capping, cyclization, and dimerization enhance peptide stability and reduce toxicity. For instance, to improve the hemolytic efficiency of the temporin peptide derived from the *Rana temporaria* frog, modifications were made by initially constructing the peptide temporin and subsequently adding two lysine residues at the N-terminus. Similarly, peptides AaeAP1 and AaeAP2 purified from *Androctonus aeneas* scorpion venom were optimized by increasing their net positive charge, thereby enhancing their interaction with microbial cell membranes while minimizing toxicity to normal cells [52]. Various modifications have also been applied to LyeTxI-b, derived from the *Lycosa erythrognatha* spider, including the removal of an amino acid group from its N-terminus followed by acylation, resulting in enhanced peptide activity [171]. Additionally, modifications to the omwaprin peptide, obtained from a snake, have led to the development of more effective versions known as omw1 and omw2. These modified peptide forms hold promise for advancing medical applications, such as using modified melittin as a coating for contact lenses to inhibit microbial replication [172]. Furthermore, conjugation with polyethylene glycol (PEGylation) and encapsulation in nanoparticles have been explored as delivery systems to improve stability, control release, and minimize off-target effects [173]. A deeper understanding of the mechanisms of action, particularly through detailed membrane interaction studies, guides the rational design of more selective peptides [174]. Moreover, combination therapies with conventional antibiotics may reduce the required peptide dose, limiting toxicity while boosting efficacy [13]. Localized applications such as coating medical devices or incorporating peptides into wound dressings allow the antimicrobial action to remain confined, minimizing harm to surrounding tissues [175].

### 5.3. Production and Purity

A significant obstacle in the development of venom-derived antimicrobial agents lies in the complexities associated with their production and purity. Natural venom is a heterogeneous mixture composed of dozens to hundreds of biologically active peptides and proteins, many of which exist in very low concentrations [176]. Isolating a specific antimicrobial peptide from this complex matrix often requires extensive fractionation and purification processes, which are labor-intensive, time-consuming, and yield limited quantities of the desired compound. Moreover, venom components often belong to large polygenic families, making it difficult to isolate a single isoform in sufficient purity and quantity for downstream applications. Traditional purification techniques such as multi-step chromatography can struggle to achieve the necessary resolution, leading to batch variability and cross-contamination with other toxins [177]. To overcome these limitations, heterologous expression systems have gained attention as promising alternatives, enabling the recombinant production of venom peptides in bacterial, yeast, insect, or mammalian cells [178]. However, these systems are not without their own challenges, including incorrect peptide folding, lack of post-translational modifications, and inclusion body formation, which may affect biological activity. Furthermore, maintaining bioactivity while achieving high expression levels and ensuring scalability for clinical development remains a persistent hurdle. Efforts to optimize codon usage, engineer fusion proteins for better solubility, or use synthetic biology approaches to design expression-compatible genes are currently underway to overcome these technical barriers [179].

Overcoming the barriers of toxicity, immunogenicity, and production scalability will be key to unlocking the clinical potential of venom-derived AMPs. The future of venom-derived AMPs lies in addressing the outlined challenges through interdisciplinary approaches. Comprehensive in vivo studies are needed to validate the efficacy and safety of modified peptides and delivery systems. Additionally, advancements in high-throughput screening and computational modeling can accelerate the identification of novel AMPs with optimal therapeutic profiles.

## 6. Conclusions

Exploring animal venoms and their peptides offers a promising frontier against antimicrobial resistance. The diverse range of bioactive compounds in these venoms presents a rich resource for developing novel anti-microbial agents. Advances in peptide modification techniques hold great potential for enhancing peptide activity while minimizing adverse effects. By coupling the synergistic impact of venom peptides and conventional antibiotics, innovative combination therapies are being developed to combat multidrug-resistant pathogens. However, challenges such as peptide stability and safety profiles underscore the importance of ongoing research and validation efforts. Moving forward, interdisciplinary collaboration and rigorous clinical evaluation will be vital for translating these promising pre-clinical discoveries into effective clinical treatments, thereby shaping the future landscape of anti-microbial therapy.

## Figures and Tables

**Table 1:** FDA-approved and clinical trial venom-based drugs: toxin sources and therapeutic applications.

Drug Name	Venom Source	Medical Use	Biological Target	FDA Approval Year	Ref.
Captopril	*Bothrops jararaca* (Brazilian pit viper)	Hypertension	Inhibition of angiotensin-converting enzyme (ACE)	1981	[27]
Eptifibatide	*Sistrurus miliarius* (Pygmy rattlesnake)	Acute coronary syndrome	Antagonist of the platelet receptor glycoprotein IIb/IIIa receptor	1998	[28]
Tirofiban	*Echis carinatus* (Saw-scaled viper)	Acute coronary syndrome	Reversible antagonist of the platelet glycoprotein IIb/IIIa receptor	1998	[29]
Ziconotide	*Conus magus* (Cone snail)	Severe chronic pain	Block N-type calcium channels	2004	[30]
Exenatide	*Heloderma suspectum* (Gila monster)	Type 2 diabetes mellitus	Binding and activation of GLP-1 receptor to reduce plasma glucose and lower HbA1c	2005	[31]
Dalazatide (ShK-186)	*Stichodactyla helianthus* (Sea anemone)	Autoimmune diseases (psoriasis, MS)	Kv1.3 potassium channel	In clinical trials	[32]
Hi1a	*Hadronyche infensa* (Australian funnel-web spider)	Cardioprotection during heart attack and stroke	Acid-sensing ion channel 1a (ASIC1a)	In clinical trials	[33]
Chlorotoxin	*Leiurus quinquestriatus* (Deathstalker scorpion)	Tumor imaging, glioma targeting	Matrix metalloproteinase-2 (MMP-2), annexin A2, and chloride channels	In clinical trials	[34]

**Table 2:** Overview of antimicrobial agents reported from animal venoms.

Animal Source	Species	Active Component	Activity Type	Target Pathogens	Ref.
Snake	*Ophiophagus hannah*	L-amino acid oxidase (L-AAO)	Antibacterial, antifungal	*Staphylococcus aureus*, *Staphylococcus epidermidis*	[35]
	*Crotalus durissus terrificus*	Crotamine	Antibacterial, antifungal, antiparasitic	*Escherichia coli*, *Bacillus subtilis*, *Candida* spp., *Plasmodium falciparum*	[36,37]
	*Hydrophis cyanocinctus*	Cathelicidin (Hc-CATH)	Antibacterial	*Staphylococcus aureus*, *Escherichia coli*, *Vibrio cholerae*	[38,39]
	*Bungarus fasciatus*	Cathelicidin-BF	Antibacterial	*Propionibacterium acnes*, *Klebsiella pneumoniae*, *Salmonella typhimurium*	[40,41]
	*Bothrops marajoensis*	L-amino acid oxidase (L-AAO)	Antibacterial, antifungal, anti-parasitic	*Staphylococcus aureus*, *Pseudomonas aeruginosa*, *Candida albicans*, *Leishmania chagasi*, *Leishmania amazonensis*	[15]
	*Naja naja*	PLA_2_ (NN-XIb-PLA_2_)	Antibacterial	*Staphylococcus aureus*, *Bacillus subtilis*	[42]
	*Oxyuranus microlepidotus*	Omwaprin	Antibacterial	*Bacillus megaterium*, *Staphylococcus warneri*	[43]
	*Crotalus adamanteus*	PLA_2_ (CaTx-II)	Antibacterial	*Burkholderia pseudomallei*, *Staphylococcus aureus*, *Enterobacter aerogenes*	[44]
Scorpion	*Lychas mucronatus*	Mucroporin-M1	Antibacterial, antiviral	*Staphylococcus aureus*, *Escherichia coli*, *Klebsiella pneumoniae*, *Pseudomonas aeruginosa*, *measles*, severe acute respiratory syndrome coronavirus (SARS-CoV), hepatitis B virus (HBV), and influenza H5N1	[45,46,47]
	*Pandinus imperator*	Pandinin peptides	Antifungal	*Candida albicans*	[48]
	*Mesobuthus martensii*	BmKn2, BmKn2-7	Antibacterial, antiviral	*Staphylococcus aureus*, *Micrococcus luteus*, *Escherichia coli*, *Pseudomonas aeruginosa*, *Neisseria gonorrhoeae*, HIV-1	[49,50,51]
	*Androctonus aeneas*	AaeAP1, AaeAP2	Antibacterial, antifungal	*Staphylococcus aureus*, *Candida albicans*	[52]
	*Isometrus scorpion*	Imcroporin	Antibacterial	*Methicillin-resistant Staphylococcus aureus*	[53]
Spider	*Lycosa singoriensis*	M-lycotoxin-Ls3a	Antibacterial, antifungal	*Staphylococcus aureus*, *Bacillus subtilis*, *Escherichia coli*, *Pseudomonas aeruginosa*, *Candida albicans*	[54]
	*Hogna carolinensis*	M-lycotoxin-Hc1a	Antibacterial, antifungal	*Staphylococcus aureus*, *Bacillus subtilis*, *Candida albicans*	[55]
	*Loxosceles gaucho*	U1-SCRTX-Lg1a	Antibacterial	*Escherichia coli*, *Pseudomonas aeruginosa*, *Enterobacter cloacae*	[56]
Honeybee	*Apis mellifera*	Melittin and PLA_2_	Antibacterial, antifungal, antiviral	Methicillin-resistant *Staphylococcus aureus*, *Escherichia coli*, *Acinetobacter baumannii*, *Candida albicans*, Herpes simplex virus (HSV), Human immunodeficiency virus (HIV), severe acute respiratory syndrome coronavirus 2 (SARS-CoV-2),	[57,58,59,60,61,62,63]
	*Apis cerana*	PLA_2_	Antibacterial	*Escherichia coli*	[64]
Wasp	*Vespa orientalis*	Whole venom	Antibacterial	*Bacillus cereus*, *Staphylococcus aureus*	[65]
	*Vespa basalis*	Mastoparan B	Antibacterial	*Enterococcus faecalis*, *Bacillus subtilis*, *Shigella flexneri*	[66]
	*Polybia paulista*	Polybia-CP	Antibacterial	*Staphylococcus aureus*, *Bacillus subtilis*, *Escherichia coli*, *Pseudomonas aeruginosa*, *Staphylococcus epidermidis*	[67,68]
Ant	*Solenopsis invicta*	Solenopsins	Antibacterial, antifungal	*Streptococcus pneumoniae*, *Staphylococcus aureus*, *Enterococcus faecalis*, *Stenotrophomonas maltophilia*, *Candida auris*	[69,70]
	*Pachycondyla goeldii*	Ponericins	Antibacterial	*Geobacillus stearothermophilus*, *Bacillus subtilis*, *Bacillus megaterium*, *Lactococcus lactis*, *Pseudomonas aeruginosa*	[71]
	*Tetramorium bicarinatum*	Bicarinalin	Antibacterial, antifungal, antiparasitic	*Cronobacter sakazakii*, *Helicobacter pylori*, *Candida albicans*, *Aspergillus niger*, *Leishmania infantum*	[72,73]

**Table 3:** Active peptide sequences of snake venom-derived cathelicidins with antimicrobial activity.

Cathelicidin Name	Snake Species	Active Peptide Sequence	Number of Residues (aa)	Ref.
NA-CATH	*Naja atra*	KRFKKFFKKLKNSVKKRAKKFFKKPKVIGVTFPF	34	[79]
OH-CATH	*Ophiophagus hannah*	KRFKKFFKKLKNSVKKRAKKFFKKPRVIGVSIPF	34	[80]
Cathelicidin-BF	*Bungarus fasciatus*	KFFRKLKKSVKKRAKEFFKKPRVIGVSIPF	30	[40]
OH-CATH30	*Ophiophagus hannah*	KFFKKLKNSVKKRAKKFFKKPRVIGVSIPF	30	[81]
Hc-CATH	*Hydrophis cyanocinctus*	KFFKRLLKSVRRAVKKFRKKPRLIGLSTLL	30	[38]
SA-CATH	*Sinonatrix annularis*	KFFKKLKKSVKKHVKKFFKKPKVIGVSIPF	30	[82]
CATHPb1	*Python bivittatus*	KRFKKFFRKIKKGFRKIFKKTKIFIGGTIPI	31	[83]
Batroxicidin (BatxC)	*Bothrops atrox*	KRFKKFFKKLKNSVKKRVKKFFRKPRVIGVTFPF	34	[84]
Crotalicidin (Ctn)	*Crotalus durissus terrificus*	KRFKKFFKKVKKSVKKRLKKIFKKPMVIGVTIPF	34	[85]
Pt_CRAMP1	*Pseudonaja textilis*	KRFKKFFMKLKKSVKKRVMKFFKKPMVIGVTFPF	34	[84]
Pt_CRAMP2	*Pseudonaja textilis*	KRFKKFFRKLKKSVKKRVKKFFKKPRVIGVTIPF	34	[84]

**Table 4:** Comparative minimum inhibitory concentration (MIC) analysis of venom-derived peptides and conventional antibiotics.

Venom-Derived Peptide	Source	Target Microbe	MIC (μg/mL)	Conventional Antibiotic	MIC (μg/mL)	Notes	Ref.
Melittin	Honeybee (*Apis mellifera*)	*Staphylococcus aureus*	2–10	Vancomycin	1–2	Disrupts bacterial membranes vs. cell wall synthesis inhibition.	[158,159]
Cathelicidin-BF	Banded krait (*Bungarus fasciatus*)	*Escherichia coli*	5–20	Ciprofloxacin	0.1–1	Membrane permeabilization vs. DNA gyrase inhibition.	[41,160]
Hadrurin	Scorpion (*Hadrurus aztecus*)	*Pseudomonas aeruginosa*	10–50	Ceftazidime	2–8	Pore formation vs. cell wall synthesis inhibition.	[144,159]
Cardiotoxin 1	Chinese cobra (*Naja atra*)	*Candida albicans*	6–50	Fluconazole	1–4	Generates ROS vs. ergosterol synthesis inhibition.	[161,162]

## Data Availability

No new data were created or analyzed in this study.

## Abbreviations

The following abbreviations are used in this manuscript:

*Staphylococcus aureus*	*S. aureus*
*Staphylococcus epidermidis*	*S. epidermidis*
*Pseudomonas aeruginosa*	*P. aeruginosa*
*Klebsiella pneumoniae*	*K. pneumoniae*
*Escherichia coli*	*E. coli*
*Bacillus cereus*	*B. cereus*
*Plasmodium berghei*	*P. berghei*
*Plasmodium falciparum*	*P. falciparum*
*Bacillus subtilis*	*B. subtilis*
*Bee Venom*	*BV*
*Helicobacter pylori*	*H. pylori*
*Candida auris*	*C. auris*

## References

[1] Baker R.E., Mahmud A.S., Miller I.F., Rajeev M., Rasambainarivo F., Rice B.L., Takahashi S., Tatem A.J., Wagner C.E., Wang L.-F. (2022). Infectious disease in an era of global change. Nat. Rev. Microbiol..

[2] Salam M.A., Al-Amin M.Y., Salam M.T., Pawar J.S., Akhter N., Rabaan A.A., Alqumber M.A.A. (2023). Antimicrobial Resistance: A Growing Serious Threat for Global Public Health. Healthcare.

[3] Murray C.J.L., Ikuta K.S., Sharara F., Swetschinski L., Robles Aguilar G., Gray A., Han C., Bisignano C., Rao P., Wool E. (2022). Global burden of bacterial antimicrobial resistance in 2019: A systematic analysis. Lancet.

[4] Gulumbe B.H., Sahal M.R., Abdulrahim A., Faggo A.A., Yusuf Z.M., Sambo K.H., Usman N.I., Bagwai M.A., Muhammad W.N., Adamu A. (2023). Antibiotic resistance and the COVID-19 pandemic: A dual crisis with complex challenges in LMICs. Health Sci. Rep..

[5] Liu B., Pop M. (2009). ARDB—Antibiotic Resistance Genes Database. Nucleic Acids Res..

[6] Woodman M.E., Worth R.G., Wooten R.M. (2012). Capsule influences the deposition of critical complement C3 levels required for the killing of Burkholderia pseudomallei via NADPH-oxidase induction by human neutrophils. PLoS ONE.

[7] Nelson M., Nunez A., Ngugi S.A., Sinclair A., Atkins T.P. (2015). Characterization of lesion formation in marmosets following inhalational challenge with different strains of Burkholderia pseudomallei. Int. J. Exp. Pathol..

[8] Lowy F.D. (1998). Staphylococcus aureus infections. N. Engl. J. Med..

[9] Guo J., Zhang H., Lin W., Lu L., Su J., Chen X. (2023). Signaling pathways and targeted therapies for psoriasis. Signal Transduct. Target. Ther..

[10] Ko S.J., Park E., Asandei A., Choi J.Y., Lee S.C., Seo C.H., Luchian T., Park Y. (2020). Bee venom-derived antimicrobial peptide melectin has broad-spectrum potency, cell selectivity, and salt-resistant properties. Sci. Rep..

[11] Utkin Y.N. (2015). Animal venom studies: Current benefits and future developments. World J. Biol. Chem..

[12] Bordon K.d.C.F., Cologna C.T., Fornari-Baldo E.C., Pinheiro-Júnior E.L., Cerni F.A., Amorim F.G., Anjolette F.A.P., Cordeiro F.A., Wiezel G.A., Cardoso I.A. (2020). From Animal Poisons and Venoms to Medicines: Achievements, Challenges and Perspectives in Drug Discovery. Front. Pharmacol..

[13] Yacoub T., Rima M., Karam M., Fajloun J. (2020). Antimicrobials from Venomous Animals: An Overview. Molecules.

[14] Perumal Samy R., Stiles B.G., Franco O.L., Sethi G., Lim L.H.K. (2017). Animal venoms as antimicrobial agents. Biochem. Pharmacol..

[15] Costa Torres A.F., Dantas R.T., Toyama M.H., Diz Filho E., Zara F.J., Rodrigues de Queiroz M.G., Pinto Nogueira N.A., Rosa de Oliveira M., de Oliveira Toyama D., Monteiro H.S. (2010). Antibacterial and antiparasitic effects of Bothrops marajoensis venom and its fractions: Phospholipase A2 and L-amino acid oxidase. Toxicon.

[16] de Oliveira A.N., Soares A.M., da Silva S.L. (2023). Peptides from Animal Venom and Poisons. Int. J. Pept. Res. Ther..

[17] A. Al-Shammery K., Hozzein W.N. (2022). Antibacterial activities of two potential peptides extracted from Polistes wattii Cameron, 1900 (Vespidae: Polistinae) wasp venom collected at Eastern Province, Saudi Arabia. PLoS ONE.

[18] Benfield A.H., Henriques S.T. (2020). Mode-of-Action of Antimicrobial Peptides: Membrane Disruption vs. Intracellular Mechanisms. Front. Med. Technol..

[19] Sabapathy T., Deplazes E., Mancera R.L. (2020). Revisiting the Interaction of Melittin with Phospholipid Bilayers: The Effects of Concentration and Ionic Strength. Int. J. Mol. Sci..

[20] Beschiaschvili G., Seelig J. (1990). Melittin binding to mixed phosphatidylglycerol/phosphatidylcholine membranes. Biochemistry.

[21] Marte F., Sankar P., Patel P., Cassagnol M. (2024). Captopril. StatPearls.

[22] Kumar A., Herrmann H.C. (1997). Tirofiban: An investigational platelet glycoprotein IIb/IIIa receptor antagonist. Expert. Opin. Investig. Drugs.

[23] Bansal A.B., Sattar Y., Patel P., Jamil R.T. (2024). Eptifibatide. StatPearls.

[24] Vu T.T., Stafford A.R., Leslie B.A., Kim P.Y., Fredenburgh J.C., Weitz J.I. (2013). Batroxobin binds fibrin with higher affinity and promotes clot expansion to a greater extent than thrombin. J. Biol. Chem..

[25] Bray G.M. (2006). Exenatide. Am. J. Health Syst. Pharm..

[26] Wie C.S., Derian A. (2024). Ziconotide. StatPearls.

[27] Ram C.V. (1982). Captopril. Arch. Intern. Med..

[28] USDA Drug Approval Package: Integrilin (Eptifibatide) NDA #20-718. https://www.accessdata.fda.gov/drugsatfda_docs/nda/98/20718_Integrilin.cfm.

[29] USDA Drug Approval Package: Aggrastat (Tirofiban Hydrochloride) NDA 20-912. https://www.accessdata.fda.gov/drugsatfda_docs/nda/99/20912S001_Aggrastat.cfm.

[30] USDA Drug Approval Package: Prialt (Ziconotide Intrathecal Infusion) NDA 21-060. https://www.accessdata.fda.gov/drugsatfda_docs/nda/2004/21-060_Prialt.cfm.

[31] USDA Drug Approval Package: Byetta (Exenatide) NDA #021773. https://www.accessdata.fda.gov/drugsatfda_docs/nda/2005/021773_byettatoc.cfm.

[32] Tarcha E.J., Olsen C.M., Probst P., Peckham D., Muñoz-Elías E.J., Kruger J.G., Iadonato S.P. (2017). Safety and pharmacodynamics of dalazatide, a Kv1.3 channel inhibitor, in the treatment of plaque psoriasis: A randomized phase 1b trial. PLoS ONE.

[33] Chassagnon I.R., McCarthy C.A., Chin Y.K., Pineda S.S., Keramidas A., Mobli M., Pham V., De Silva T.M., Lynch J.W., Widdop R.E. (2017). Potent neuroprotection after stroke afforded by a double-knot spider-venom peptide that inhibits acid-sensing ion channel 1a. Proc. Natl. Acad. Sci. USA.

[34] Dardevet L., Rani D., Aziz T.A., Bazin I., Sabatier J.M., Fadl M., Brambilla E., De Waard M. (2015). Chlorotoxin: A helpful natural scorpion peptide to diagnose glioma and fight tumor invasion. Toxins.

[35] Lee M.L., Tan N.H., Fung S.Y., Sekaran S.D. (2011). Antibacterial action of a heat-stable form of L-amino acid oxidase isolated from king cobra (*Ophiophagus hannah*) venom. Comp. Biochem. Physiol. C Toxicol. Pharmacol..

[36] Yamane E.S., Bizerra F.C., Oliveira E.B., Moreira J.T., Rajabi M., Nunes G.L., de Souza A.O., da Silva I.D., Yamane T., Karpel R.L. (2013). Unraveling the antifungal activity of a South American rattlesnake toxin crotamine. Biochimie.

[37] El Chamy Maluf S., Dal Mas C., Oliveira E.B., Melo P.M., Carmona A.K., Gazarini M.L., Hayashi M.A. (2016). Inhibition of malaria parasite Plasmodium falciparum development by crotamine, a cell penetrating peptide from the snake venom. Peptides.

[38] Wei L., Gao J., Zhang S., Wu S., Xie Z., Ling G., Kuang Y.-Q., Yang Y., Yu H., Wang Y. (2015). Identification and Characterization of the First Cathelicidin from Sea Snakes with Potent Antimicrobial and Anti-inflammatory Activity and Special Mechanism. J. Biol. Chem..

[39] Carlile S.R., Shiels J., Kerrigan L., Delaney R., Megaw J., Gilmore B.F., Weldon S., Dalton J.P., Taggart C.C. (2019). Sea snake cathelicidin (Hc-cath) exerts a protective effect in mouse models of lung inflammation and infection. Sci. Rep..

[40] Wang Y., Hong J., Liu X., Yang H., Liu R., Wu J., Wang A., Lin D., Lai R. (2008). Snake Cathelicidin from Bungarus fasciatus Is a Potent Peptide Antibiotics. PLoS ONE.

[41] Wang Y., Zhang Z., Chen L., Guang H., Li Z., Yang H., Li J., You D., Yu H., Lai R. (2011). Cathelicidin-BF, a Snake Cathelicidin-Derived Antimicrobial Peptide, Could Be an Excellent Therapeutic Agent for Acne Vulgaris. PLoS ONE.

[42] Sudarshan S., Dhananjaya B.L. (2016). Antibacterial activity of an acidic phospholipase A2 (NN-XIb-PLA2) from the venom of Naja naja (*Indian cobra*). Springerplus.

[43] Nair D.G., Fry B.G., Alewood P., Kumar P.P., Kini R.M. (2007). Antimicrobial activity of omwaprin, a new member of the waprin family of snake venom proteins. Biochem. J..

[44] Samy R.P., Kandasamy M., Gopalakrishnakone P., Stiles B.G., Rowan E.G., Becker D., Shanmugam M.K., Sethi G., Chow V.T. (2014). Wound healing activity and mechanisms of action of an antibacterial protein from the venom of the eastern diamondback rattlesnake (*Crotalus adamanteus*). PLoS ONE.

[45] Dai C., Ma Y., Zhao Z., Zhao R., Wang Q., Wu Y., Cao Z., Li W. (2008). Mucroporin, the first cationic host defense peptide from the venom of Lychas mucronatus. Antimicrob. Agents Chemother..

[46] Li Q., Zhao Z., Zhou D., Chen Y., Hong W., Cao L., Yang J., Zhang Y., Shi W., Cao Z. (2011). Virucidal activity of a scorpion venom peptide variant mucroporin-M1 against measles, SARS-CoV and influenza H5N1 viruses. Peptides.

[47] Zhao Z., Hong W., Zeng Z., Wu Y., Hu K., Tian X., Li W., Cao Z. (2012). Mucroporin-M1 inhibits hepatitis B virus replication by activating the mitogen-activated protein kinase (MAPK) pathway and down-regulating HNF4α in vitro and in vivo. J. Biol. Chem..

[48] Zeng X.-C., Zhou L., Shi W., Luo X., Zhang L., Nie Y., Wang J., Wu S., Cao B., Cao H. (2013). Three new antimicrobial peptides from the scorpion Pandinus imperator. Peptides.

[49] Zeng X.C., Wang S.X., Zhu Y., Zhu S.Y., Li W.X. (2004). Identification and functional characterization of novel scorpion venom peptides with no disulfide bridge from Buthus martensii Karsch. Peptides.

[50] Cao L., Dai C., Li Z., Fan Z., Song Y., Wu Y., Cao Z., Li W. (2012). Antibacterial activity and mechanism of a scorpion venom peptide derivative in vitro and in vivo. PLoS ONE.

[51] Chen Y., Cao L., Zhong M., Zhang Y., Han C., Li Q., Yang J., Zhou D., Shi W., He B. (2012). Anti-HIV-1 activity of a new scorpion venom peptide derivative Kn2-7. PLoS ONE.

[52] Du Q., Hou X., Wang L., Zhang Y., Xi X., Wang H., Zhou M., Duan J., Wei M., Chen T. (2015). AaeAP1 and AaeAP2: Novel Antimicrobial Peptides from the Venom of the Scorpion, Androctonus aeneas: Structural Characterisation, Molecular Cloning of Biosynthetic Precursor-Encoding cDNAs and Engineering of Analogues with Enhanced Antimicrobial and Anticancer Activities. Toxins.

[53] Zhao Z., Ma Y., Dai C., Zhao R., Li S., Wu Y., Cao Z., Li W. (2009). Imcroporin, a new cationic antimicrobial peptide from the venom of the scorpion Isometrus maculates. Antimicrob. Agents Chemother..

[54] Budnik B.A., Olsen J.V., Egorov T.A., Anisimova V.E., Galkina T.G., Musolyamov A.K., Grishin E.V., Zubarev R.A. (2004). De novo sequencing of antimicrobial peptides isolated from the venom glands of the wolf spider Lycosa singoriensis. J. Mass. Spectrom..

[55] Yan S., Wu G. (2012). Detailed folding structures of M-lycotoxin-Hc1a and its mutageneses using 2D HP model. Mol. Simul..

[56] Segura-Ramírez P.J., Silva Júnior P.I. (2018). Loxosceles gaucho Spider Venom: An Untapped Source of Antimicrobial Agents. Toxins.

[57] Choi J.H., Jang A.Y., Lin S., Lim S., Kim D., Park K., Han S.M., Yeo J.H., Seo H.S. (2015). Melittin, a honeybee venom-derived antimicrobial peptide, may target methicillin-resistant Staphylococcus aureus. Mol. Med. Rep..

[58] Zhang H.-Q., Sun C., Xu N., Liu W. (2024). The current landscape of the antimicrobial peptide melittin and its therapeutic potential. Front. Immunol..

[59] Baghian A., Jaynes J., Enright F., Kousoulas K.G. (1997). An amphipathic alpha-helical synthetic peptide analogue of melittin inhibits herpes simplex virus-1 (HSV-1)-induced cell fusion and virus spread. Peptides.

[60] Wachinger M., Kleinschmidt A., Winder D., von Pechmann N., Ludvigsen A., Neumann M., Holle R., Salmons B., Erfle V., Brack-Werner R. (1998). Antimicrobial peptides melittin and cecropin inhibit replication of human immunodeficiency virus 1 by suppressing viral gene expression. J. Gen. Virol..

[61] Al-Rabia M.W., Alhakamy N.A., Ahmed O.A.A., Eljaaly K., Alaofi A.L., Mostafa A., Asfour H.Z., Aldarmahi A.A., Darwish K.M., Ibrahim T.S. (2021). Repurposing of Sitagliptin- Melittin Optimized Nanoformula against SARS-CoV-2: Antiviral Screening and Molecular Docking Studies. Pharmaceutics.

[62] Askari P., Namaei M.H., Ghazvini K., Hosseini M. (2021). In vitro and in vivo toxicity and antibacterial efficacy of melittin against clinical extensively drug-resistant bacteria. BMC Pharmacol. Toxicol..

[63] Leandro L.F., Mendes C.A., Casemiro L.A., Vinholis A.H., Cunha W.R., de Almeida R., Martins C.H. (2015). Antimicrobial activity of apitoxin, melittin and phospholipase A_2_ of honey bee (*Apis mellifera*) venom against oral pathogens. An. Acad. Bras. Cienc..

[64] Indria Puti Mustika K.L.M.S.B.W.G. (2022). Isolation and Antibacterial Activity of Honey Bee Venom Bioactive from Apis cerana. Int. J. Technol..

[65] Farag R., Swaby S. (2018). Antimicrobial effects of wasp (*Vespa orientalis*) venom. Egypt. Pharm. J..

[66] Park N.G., Yamato Y., Lee S., Sugihara G. (1995). Interaction of mastoparan-B from venom of a hornet in Taiwan with phospholipid bilayers and its antimicrobial activity. Biopolymers.

[67] Souza B.M., Mendes M.A., Santos L.D., Marques M.R., César L.M., Almeida R.N., Pagnocca F.C., Konno K., Palma M.S. (2005). Structural and functional characterization of two novel peptide toxins isolated from the venom of the social wasp Polybia paulista. Peptides.

[68] Wang K., Yan J., Chen R., Dang W., Zhang B., Zhang W., Song J., Wang R. (2012). Membrane-active action mode of polybia-CP, a novel antimicrobial peptide isolated from the venom of Polybia paulista. Antimicrob. Agents Chemother..

[69] Sullivan D.C., Flowers H., Rockhold R., Herath H.M.T.B., Nanayakkara N.P.D. (2009). Antibacterial Activity of Synthetic Fire Ant Venom: The Solenopsins and Isosolenopsins. Am. J. Med. Sci..

[70] Honorato L., Artunduaga Bonilla J.J., Ribeiro da Silva L., Kornetz J., Zamith-Miranda D., Valdez A.F., Nosanchuk J.D., Gonçalves Paterson Fox E., Nimrichter L. (2024). Alkaloids solenopsins from fire ants display in vitro and in vivo activity against the yeast Candida auris. Virulence.

[71] Orivel J., Redeker V., Le Caer J.-P., Krier F., Revol-Junelles A.-M., Longeon A., Chaffotte A., Dejean A., Rossier J. (2001). Ponericins, New Antibacterial and Insecticidal Peptides from the Venom of the Ant *Pachycondyla goeldii*. J. Biol. Chem..

[72] Rifflet A., Gavalda S., Téné N., Orivel J., Leprince J., Guilhaudis L., Génin E., Vétillard A., Treilhou M. (2012). Identification and characterization of a novel antimicrobial peptide from the venom of the ant Tetramorium bicarinatum. Peptides.

[73] Téné N., Bonnafé E., Berger F., Rifflet A., Guilhaudis L., Ségalas-Milazzo I., Pipy B., Coste A., Leprince J., Treilhou M. (2016). Biochemical and biophysical combined study of bicarinalin, an ant venom antimicrobial peptide. Peptides.

[74] Munawar A., Ali S.A., Akrem A., Betzel C. (2018). Snake Venom Peptides: Tools of Biodiscovery. Toxins.

[75] Perumal Samy R., Gopalakrishnakone P., Ho B., Chow V.T. (2008). Purification, characterization and bactericidal activities of basic phospholipase A2 from the venom of *Agkistrodon halys* (Chinese pallas). Biochimie.

[76] Abdullahi Z.U., Musa S.S., Abu-Odah H., Ahmed A., Lawan A.A., Bello U.M. (2023). Bactericidal Effects of Snake Venom Phospholipases A2: A Systematic Review and Analysis of Minimum Inhibitory Concentration. Physiologia.

[77] Xu S., Gu L., Jiang T., Zhou Y., Lin Z. (2003). Structures of cadmium-binding acidic phospholipase A2 from the venom of Agkistrodon halys Pallas at 1.9Å resolution. Biochem. Biophys. Res. Commun..

[78] de Barros E., Gonçalves R.M., Cardoso M.H., Santos N.C., Franco O.L., Cândido E.S. (2019). Snake Venom Cathelicidins as Natural Antimicrobial Peptides. Front. Pharmacol..

[79] Du H., Samuel R.L., Massiah M.A., Gillmor S.D. (2015). The structure and behavior of the NA-CATH antimicrobial peptide with liposomes. Biochim. Et Biophys. Acta (BBA)-Biomembr..

[80] Zhao H., Gan T.-X., Liu X.-D., Jin Y., Lee W.-H., Shen J.-H., Zhang Y. (2008). Identification and characterization of novel reptile cathelicidins from elapid snakes. Peptides.

[81] Li S.A., Lee W.H., Zhang Y. (2012). Efficacy of OH-CATH30 and its analogs against drug-resistant bacteria in vitro and in mouse models. Antimicrob. Agents Chemother..

[82] Wang A., Zhang F., Guo Z., Chen Y., Zhang M., Yu H., Wang Y. (2019). Characterization of a Cathelicidin from the Colubrinae Snake, Sinonatrix annularis. Zool. Sci..

[83] Cai S., Qiao X., Feng L., Shi N., Wang H., Yang H., Guo Z., Wang M., Chen Y., Wang Y. (2018). Python Cathelicidin CATHPb1 Protects against Multidrug-Resistant Staphylococcal Infections by Antimicrobial-Immunomodulatory Duality. J. Med. Chem..

[84] Falcao C.B., de La Torre B.G., Pérez-Peinado C., Barron A.E., Andreu D., Rádis-Baptista G. (2014). Vipericidins: A novel family of cathelicidin-related peptides from the venom gland of South American pit vipers. Amino Acids.

[85] Falcao C.B., Pérez-Peinado C., de la Torre B.G., Mayol X., Zamora-Carreras H., Jiménez M., Rádis-Baptista G., Andreu D. (2015). Structural Dissection of Crotalicidin, a Rattlesnake Venom Cathelicidin, Retrieves a Fragment with Antimicrobial and Antitumor Activity. J. Med. Chem..

[86] Quintana J.C., Chacón A.M., Vargas L., Segura C., Gutiérrez J.M., Alarcón J.C. (2012). Antiplasmodial effect of the venom of Crotalus durissus cumanensis, crotoxin complex and Crotoxin B. Acta Trop..

[87] Banigan J.R., Mandal K., Sawaya M.R., Thammavongsa V., Hendrickx A.P., Schneewind O., Yeates T.O., Kent S.B. (2010). Determination of the X-ray structure of the snake venom protein omwaprin by total chemical synthesis and racemic protein crystallography. Protein Sci..

[88] Rádis-Baptista G., Moreno F.B.M.B., Nogueira L.d.L., Martins A.M.C., Toyama D.d.O., Toyama M.H., Cavada B.S., de Azevedo W.F., Yamane T. (2006). Crotacetin, a novel snake venom C-type lectin homolog of convulxin, exhibits an unpredictable antimicrobial activity. Cell Biochem. Biophys..

[89] Ullah A., Souza T.A., Abrego J.R., Betzel C., Murakami M.T., Arni R.K. (2012). Structural insights into selectivity and cofactor binding in snake venom L-amino acid oxidases. Biochem. Biophys. Res. Commun..

[90] Sulca M.A., Remuzgo C., Cárdenas J., Kiyota S., Cheng E., Bemquerer M.P., Machini M.T. (2017). Venom of the Peruvian snake Bothriopsis oligolepis: Detection of antibacterial activity and involvement of proteolytic enzymes and C-type lectins in growth inhibition of Staphylococcus aureus. Toxicon.

[91] Okumu M.O., Eyaan K.L., Bett L.K., Gitahi N. (2023). Antibacterial Activity of Venom from the Puff Adder (*Bitis arietans*), Egyptian Cobra (*Naja haje*), and Red Spitting Cobra (*Naja pallida*). Int. J. Microbiol..

[92] Ortiz E., Gurrola G.B., Schwartz E.F., Possani L.D. (2015). Scorpion venom components as potential candidates for drug development. Toxicon.

[93] Rincón-Cortés C.A., Bayona-Rojas M.A., Reyes-Montaño E.A., Vega-Castro N.A. (2022). Antimicrobial Activity Developed by Scorpion Venoms and Its Peptide Component. Toxins.

[94] Moerman L., Bosteels S., Noppe W., Willems J., Clynen E., Schoofs L., Thevissen K., Tytgat J., Van Eldere J., Van Der Walt J. (2002). Antibacterial and antifungal properties of alpha-helical, cationic peptides in the venom of scorpions from southern Africa. Eur. J. Biochem..

[95] Bao A., Zhong J., Zeng X.-C., Nie Y., Zhang L., Peng Z.F. (2015). A novel cysteine-free venom peptide with strong antimicrobial activity against antibiotics-resistant pathogens from the scorpion Opistophthalmus glabrifrons. J. Pept. Sci..

[96] Guilhelmelli F., Vilela N., Smidt K.S., de Oliveira M.A., da Cunha Morales Álvares A., Rigonatto M.C.L., da Silva Costa P.H., Tavares A.H., Freitas S.M.d., Nicola A.M. (2016). Activity of Scorpion Venom-Derived Antifungal Peptides against Planktonic Cells of Candida spp. and Cryptococcus neoformans and Candida albicans Biofilms. Front. Microbiol..

[97] Harrison P.L., Abdel-Rahman M.A., Miller K., Strong P.N. (2014). Antimicrobial peptides from scorpion venoms. Toxicon.

[98] Gao B., Xu J., Rodriguez Mdel C., Lanz-Mendoza H., Hernández-Rivas R., Du W., Zhu S. (2010). Characterization of two linear cationic antimalarial peptides in the scorpion Mesobuthus eupeus. Biochimie.

[99] Wang X., Wang G. (2016). Insights into Antimicrobial Peptides from Spiders and Scorpions. Protein Pept. Lett..

[100] Santos D.M., Reis P.V., Pimenta A.M.C., Gopalakrishnakone P., Corzo G.A., Diego-Garcia E., de Lima M.E. (2015). Antimicrobial Peptides in Spider Venoms. Spider Venoms.

[101] Shin M.K., Hwang I.-W., Jang B.-Y., Bu K.-B., Han D.-H., Lee S.-H., Oh J.W., Yoo J.S., Sung J.-S. (2023). The Identification of a Novel Spider Toxin Peptide, Lycotoxin-Pa2a, with Antibacterial and Anti-Inflammatory Activities. Antibiotics.

[102] Shin M.K., Hwang I.-W., Jang B.-Y., Bu K.-B., Yoo J.S., Sung J.-S. (2023). In silico identification of novel antimicrobial peptides from the venom gland transcriptome of the spider Argiope bruennichi (Scopoli, 1772). Front. Microbiol..

[103] Kim W. (2021). Bee Venom and Its Sub-Components: Characterization, Pharmacology, and Therapeutics. Toxins.

[104] Wehbe R., Frangieh J., Rima M., El Obeid D., Sabatier J.-M., Fajloun Z. (2019). Bee Venom: Overview of Main Compounds and Bioactivities for Therapeutic Interests. Molecules.

[105] Yuva B. (2015). Bee Venom: Its Potential Use in Alternative Medicine. Anti-Infect. Agents.

[106] Han S.M., Kim J.M., Hong I.P., Woo S.O., Kim S.G., Jang H.R., Pak S.C. (2016). Antibacterial Activity and Antibiotic-Enhancing Effects of Honeybee Venom against Methicillin-Resistant Staphylococcus aureus. Molecules.

[107] Han S., Yeo J., Baek H., Lin S.M., Meyer S., Molan P. (2009). Postantibiotic effect of purified melittin from honeybee (Apis mellifera) venom against Escherichia coli and Staphylococcus aureus. J. Asian Nat. Prod. Res..

[108] Ramirez L.S., Pande J., Shekhtman A. (2019). Helical Structure of Recombinant Melittin. J. Phys. Chem. B.

[109] Scott D.L., Otwinowski Z., Gelb M.H., Sigler P.B. (1990). Crystal structure of bee-venom phospholipase A2 in a complex with a transition-state analogue. Science.

[110] Kuzmenkov A.I., Peigneur S., Nasburg J.A., Mineev K.S., Nikolaev M.V., Pinheiro-Junior E.L., Arseniev A.S., Wulff H., Tytgat J., Vassilevski A.A. (2022). Apamin structure and pharmacology revisited. Front. Pharmacol..

[111] Socarras K.M., Theophilus P.A.S., Torres J.P., Gupta K., Sapi E. (2017). Antimicrobial Activity of Bee Venom and Melittin against Borrelia burgdorferi. Antibiotics.

[112] Uddin M.B., Lee B.-H., Nikapitiya C., Kim J.-H., Kim T.-H., Lee H.-C., Kim C.G., Lee J.-S., Kim C.-J. (2016). Inhibitory effects of bee venom and its components against viruses in vitro and in vivo. J. Microbiol..

[113] Yu A.-R., Kim J.-J., Park G.-S., Oh S.-M., Han C.-S., Lee M.-Y. (2012). The antifungal activity of bee venom against dermatophytes. J. Appl. Biol. Chem..

[114] Lee S.B. (2016). Antifungal Activity of Bee Venom and Sweet Bee Venom against Clinically Isolated Candida albicans. J. Pharmacopunct..

[115] Al-Ani I., Zimmermann S., Reichling J., Wink M. (2015). Pharmacological synergism of bee venom and melittin with antibiotics and plant secondary metabolites against multi-drug resistant microbial pathogens. Phytomedicine.

[116] Luo L., Kamau P.M., Lai R. (2022). Bioactive Peptides and Proteins from Wasp Venoms. Biomolecules.

[117] Bulet P., Stöcklin R., Menin L. (2004). Anti-microbial peptides: From invertebrates to vertebrates. Immunol. Rev..

[118] Reddy K.V., Yedery R.D., Aranha C. (2004). Antimicrobial peptides: Premises and promises. Int. J. Antimicrob. Agents.

[119] Jalaei J., Fazeli M., Rajaian H., Shekarforoush S.S. (2014). In vitro antibacterial effect of wasp (*Vespa orientalis*) venom. J. Venom. Anim. Toxins Incl. Trop. Dis..

[120] Katsu T., Kuroko M., Morikawa T., Sanchika K., Yamanaka H., Shinoda S., Fujita Y. (1990). Interaction of wasp venom mastoparan with biomembranes. Biochim. Et Biophys. Acta (BBA)-Biomembr..

[121] Lin C.H., Tzen J.T., Shyu C.L., Yang M.J., Tu W.C. (2011). Structural and biological characterization of mastoparans in the venom of Vespa species in Taiwan. Peptides.

[122] Yang X., Wang Y., Lee W.H., Zhang Y. (2013). Antimicrobial peptides from the venom gland of the social wasp Vespa tropica. Toxicon.

[123] Boaro A., Ageitos L., Torres M.D.T., Blasco E.B., Oztekin S., de la Fuente-Nunez C. (2023). Structure-function-guided design of synthetic peptides with anti-infective activity derived from wasp venom. Cell Rep. Phys. Sci..

[124] Torres M.D.T., Silva A.F., Andrade G.P., Pedron C.N., Cerchiaro G., Ribeiro A.O., Oliveira V.X., de la Fuente-Nunez C. (2020). The wasp venom antimicrobial peptide polybia-CP and its synthetic derivatives display antiplasmodial and anticancer properties. Bioeng. Transl. Med..

[125] Blum M.S. (1992). Ant Venoms: Chemical and Pharmacological Properties. J. Toxicol. Toxin Rev..

[126] Xu G., Chen L. (2023). Biological Activities and Ecological Significance of Fire Ant Venom Alkaloids. Toxins.

[127] Chen L., Fadamiro H.Y. (2009). Re-investigation of venom chemistry of Solenopsis fire ants. II. Identification of novel alkaloids in S. invicta. Toxicon.

[128] Menk J.J., Matuhara Y.E., Sebestyen-França H., Henrique-Silva F., Ferro M., Rodrigues R.S., Santos-Júnior C.D. (2023). Antimicrobial Peptide Arsenal Predicted from the Venom Gland Transcriptome of the Tropical Trap-Jaw Ant Odontomachus chelifer. Toxins.

[129] Guzman J., Téné N., Touchard A., Castillo D., Belkhelfa H., Haddioui-Hbabi L., Treilhou M., Sauvain M. (2017). Anti-Helicobacter pylori Properties of the Ant-Venom Peptide Bicarinalin. Toxins.

[130] Perumal Samy R., Gopalakrishnakone P., Thwin M.M., Chow T.K., Bow H., Yap E.H., Thong T.W. (2007). Antibacterial activity of snake, scorpion and bee venoms: A comparison with purified venom phospholipase A2 enzymes. J. Appl. Microbiol..

[131] Lima W.G., de Lima M.E. (2023). Therapeutic Prospection of Animal Venoms-Derived Antimicrobial Peptides against Infections by Multidrug-Resistant Acinetobacter baumannii: A Systematic Review of Pre-Clinical Studies. Toxins.

[132] Amorim-Carmo B., Parente A.M.S., Souza E.S., Silva-Junior A.A., Araújo R.M., Fernandes-Pedrosa M.F. (2022). Antimicrobial Peptide Analogs From Scorpions: Modifications and Structure-Activity. Front. Mol. Biosci..

[133] Graf M., Wilson D.N. (2019). Intracellular Antimicrobial Peptides Targeting the Protein Synthesis Machinery. Adv. Exp. Med. Biol..

[134] Moravej H., Moravej Z., Yazdanparast M., Heiat M., Mirhosseini A., Moosazadeh Moghaddam M., Mirnejad R. (2018). Antimicrobial Peptides: Features, Action, and Their Resistance Mechanisms in Bacteria. Microb. Drug Resist..

[135] Aisenbrey C., Marquette A., Bechinger B. (2019). The Mechanisms of Action of Cationic Antimicrobial Peptides Refined by Novel Concepts from Biophysical Investigations. Adv. Exp. Med. Biol..

[136] Guryanova S.V., Ovchinnikova T.V. (2022). Immunomodulatory and Allergenic Properties of Antimicrobial Peptides. Int. J. Mol. Sci..

[137] Bocian A., Hus K.K. (2020). Antibacterial properties of snake venom components. Chem. Pap..

[138] Oguiura N., Boni-Mitake M., Affonso R., Zhang G. (2011). In vitro antibacterial and hemolytic activities of crotamine, a small basic myotoxin from rattlesnake Crotalus durissus. J. Antibiot..

[139] Santamaría C., Larios S., Angulo Y., Pizarro-Cerda J., Gorvel J.P., Moreno E., Lomonte B. (2005). Antimicrobial activity of myotoxic phospholipases A2 from crotalid snake venoms and synthetic peptide variants derived from their C-terminal region. Toxicon.

[140] Páramo L., Lomonte B., Pizarro-Cerdá J., Bengoechea J.-A., Gorvel J.-P., Moreno E. (1998). Bactericidal activity of Lys49 and Asp49 myotoxic phospholipases A2 from Bothrops asper snake venom. Eur. J. Biochem..

[141] Costa B.A., Sanches L., Gomide A.B., Bizerra F., Dal Mas C., Oliveira E.B., Perez K.R., Itri R., Oguiura N., Hayashi M.A. (2014). Interaction of the rattlesnake toxin crotamine with model membranes. J. Phys. Chem. B.

[142] Coorens M., van Dijk A., Bikker F., Veldhuizen E.J., Haagsman H.P. (2015). Importance of Endosomal Cathelicidin Degradation To Enhance DNA-Induced Chicken Macrophage Activation. J. Immunol..

[143] Nicastro G., Franzoni L., de Chiara C., Mancin A.C., Giglio J.R., Spisni A. (2003). Solution structure of crotamine, a Na+ channel affecting toxin from Crotalus durissus terrificus venom. Eur. J. Biochem..

[144] Torres-Larios A., Gurrola G.B., Zamudio F.Z., Possani L.D. (2000). Hadrurin, a new antimicrobial peptide from the venom of the scorpion Hadrurus aztecus. Eur. J. Biochem..

[145] Mandard N., Sy D., Maufrais C., Bonmatin J.M., Bulet P., Hetru C., Vovelle F. (1999). Androctonin, a novel antimicrobial peptide from scorpion Androctonus australis: Solution structure and molecular dynamics simulations in the presence of a lipid monolayer. J. Biomol. Struct. Dyn..

[146] Yan L., Adams M.E. (1998). Lycotoxins, Antimicrobial Peptides from Venom of the Wolf SpiderLycosa carolinensis *. J. Biol. Chem..

[147] Habermann E. (1972). Bee and wasp venoms. Science.

[148] Steiner H., Hultmark D., Engström A., Bennich H., Boman H.G. (1981). Sequence and specificity of two antibacterial proteins involved in insect immunity. Nature.

[149] Giacometti A., Cirioni O., Kamysz W., D’Amato G., Silvestri C., Del Prete M.S., Łukasiak J., Scalise G. (2003). Comparative activities of cecropin A, melittin, and cecropin A-melittin peptide CA(1-7)M(2-9)NH2 against multidrug-resistant nosocomial isolates of Acinetobacter baumannii. Peptides.

[150] Lee M.T., Sun T.L., Hung W.C., Huang H.W. (2013). Process of inducing pores in membranes by melittin. Proc. Natl. Acad. Sci. USA.

[151] Lima W.G., de Brito J.C.M., Cardoso V.N., Fernandes S.O.A. (2021). In-depth characterization of antibacterial activity of melittin against Staphylococcus aureus and use in a model of non-surgical MRSA-infected skin wounds. Eur. J. Pharm. Sci..

[152] Brogden K.A. (2005). Antimicrobial peptides: Pore formers or metabolic inhibitors in bacteria?. Nat. Rev. Microbiol..

[153] Benmoussa K., Authier H., Prat M., AlaEddine M., Lefèvre L., Rahabi M.C., Bernad J., Aubouy A., Bonnafé E., Leprince J. (2017). P17, an Original Host Defense Peptide from Ant Venom, Promotes Antifungal Activities of Macrophages through the Induction of C-Type Lectin Receptors Dependent on LTB4-Mediated PPARγ Activation. Front. Immunol..

[154] Cecilio A.B., Caldas S., Oliveira R.A., Santos A.S., Richardson M., Naumann G.B., Schneider F.S., Alvarenga V.G., Estevão-Costa M.I., Fuly A.L. (2013). Molecular characterization of Lys49 and Asp49 phospholipases A_2_ from snake venom and their antiviral activities against *Dengue virus*. Toxins.

[155] Fenard D., Lambeau G., Valentin E., Lefebvre J.C., Lazdunski M., Doglio A. (1999). Secreted phospholipases A(2), a new class of HIV inhibitors that block virus entry into host cells. J. Clin. Invest..

[156] El-Bitar A.M.H., Sarhan M., Abdel-Rahman M.A., Quintero-Hernandez V., Aoki-Utsubo C., Moustafa M.A., Possani L.D., Hotta H. (2020). Smp76, a Scorpine-Like Peptide Isolated from the Venom of the Scorpion Scorpio maurus palmatus, with a Potent Antiviral Activity Against Hepatitis C Virus and Dengue Virus. Int. J. Pept. Res. Ther..

[157] Ji M., Zhu T., Xing M., Luan N., Mwangi J., Yan X., Mo G., Rong M., Li B., Lai R. (2019). An Antiviral Peptide from Alopecosa nagpag Spider Targets NS2B-NS3 Protease of Flaviviruses. Toxins.

[158] Jalalifar S., Razavi S., Mirzaei R., Irajian G., Pooshang Bagheri K. (2024). A hope for ineffective antibiotics to return to treatment: Investigating the anti-biofilm potential of melittin alone and in combination with penicillin and oxacillin against multidrug resistant-MRSA and -VRSA. Front. Microbiol..

[159] Humphries R., Bobenchik A., Hindler J., Schuetz A. (2021). Overview of Changes to the Clinical and Laboratory Standards Institute Performance Standards for Antimicrobial Susceptibility Testing, M100, 31 st Edition. J. Clin. Microbiol..

[160] Giske C., Turnidge J., Canton R., Kahlmeter G. (2022). Update from the European Committee on Antimicrobial Susceptibility Testing (EUCAST). J. Clin. Microbiol..

[161] Pfaller M., Chaturvedi V., Espinel-Ingroff A., Ghannoum M., Gosey L.L., Odds F.C. (2008). Reference method for broth dilution antifungal susceptibility testing of yeasts: Approved standard-second edition. CLSI document M27-A2 (ISBN 1-56238-469-4). Clin. Lab. Stand. Inst..

[162] Sala A., Cabassi C.S., Santospirito D., Polverini E., Flisi S., Cavirani S., Taddei S. (2018). Novel Naja atra cardiotoxin 1 (CTX-1) derived antimicrobial peptides with broad spectrum activity. PLoS ONE.

[163] Garcia F., Villegas E., Espino-Solis G.P., Rodriguez A., Paniagua-Solis J.F., Sandoval-Lopez G., Possani L.D., Corzo G. (2013). Antimicrobial peptides from arachnid venoms and their microbicidal activity in the presence of commercial antibiotics. J. Antibiot..

[164] Ko S.J., Kim M.K., Bang J.K., Seo C.H., Luchian T., Park Y. (2017). Macropis fulvipes Venom component Macropin Exerts its Antibacterial and Anti-Biofilm Properties by Damaging the Plasma Membranes of Drug Resistant Bacteria. Sci. Rep..

[165] Obiang-Obounou B.W., Kang O.H., Choi J.G., Keum J.H., Kim S.B., Mun S.H., Shin D.W., Kim K.W., Park C.B., Kim Y.G. (2011). The mechanism of action of sanguinarine against methicillin-resistant Staphylococcus aureus. J. Toxicol. Sci..

[166] Bevalian P., Pashaei F., Akbari R., Pooshang Bagheri K. (2021). Eradication of vancomycin-resistant Staphylococcus aureus on a mouse model of third-degree burn infection by melittin: An antimicrobial peptide from bee venom. Toxicon.

[167] El-Seedi H., Abd El-Wahed A., Yosri N., Musharraf S.G., Chen L., Moustafa M., Zou X., Al-Mousawi S., Guo Z., Khatib A. (2020). Antimicrobial Properties of Apis mellifera’s Bee Venom. Toxins.

[168] Hakimi Alni R., Tavasoli F., Barati A., Shahrokhi Badarbani S., Salimi Z., Babaeekhou L. (2020). Synergistic activity of melittin with mupirocin: A study against methicillin-resistant S. Aureus (MRSA) and methicillin-susceptible S. Aureus (MSSA) isolates. Saudi J. Biol. Sci..

[169] Wang A., Zheng Y., Zhu W., Yang L., Yang Y., Peng J. (2022). Melittin-Based Nano-Delivery Systems for Cancer Therapy. Biomolecules.

[170] Hancock R.E.W., Sahl H.-G. (2006). Antimicrobial and host-defense peptides as new anti-infective therapeutic strategies. Nat. Biotechnol..

[171] Reis P.V.M., Boff D., Verly R.M., Melo-Braga M.N., Cortés M.E., Santos D.M., Pimenta A.M.C., Amaral F.A., Resende J.M., de Lima M.E. (2018). LyeTxI-b, a Synthetic Peptide Derived From Lycosa erythrognatha Spider Venom, Shows Potent Antibiotic Activity in Vitro and in Vivo. Front. Microbiol..

[172] Thankappan B., Angayarkanni J. (2019). Biological characterization of omw1 and omw2: Antimicrobial peptides derived from omwaprin. 3 Biotech..

[173] Fadaka A.O., Sibuyi N.R.S., Madiehe A.M., Meyer M. (2021). Nanotechnology-Based Delivery Systems for Antimicrobial Peptides. Pharmaceutics.

[174] Gagandeep K.R., Balenahalli Narasingappa R., Vishnu Vyas G. (2024). Unveiling mechanisms of antimicrobial peptide: Actions beyond the membranes disruption. Heliyon.

[175] Aburayan W.S., Alajmi A.M., Alfahad A.J., Alsharif W.K., Alshehri A.A., Booq R.Y., Alsudir S.A., Alsulaihem F.M., Bukhary H.A., Badr M.Y. (2022). Melittin from Bee Venom Encapsulating Electrospun Fibers as a Potential Antimicrobial Wound Dressing Patches for Skin Infections. Pharmaceutics.

[176] Calvete J.J., Juárez P., Sanz L. (2007). Snake venomics. Strategy and applications. J. Mass. Spectrom..

[177] Abd El-Aziz T.M., Soares A.G., Stockand J.D. (2020). Advances in venomics: Modern separation techniques and mass spectrometry. J. Chromatogr. B Anal. Technol. Biomed. Life Sci..

[178] Klint J.K., Senff S., Saez N.J., Seshadri R., Lau H.Y., Bende N.S., Undheim E.A.B., Rash L.D., Mobli M., King G.F. (2013). Production of Recombinant Disulfide-Rich Venom Peptides for Structural and Functional Analysis via Expression in the Periplasm of E. coli. PLoS ONE.

[179] Oliveira A.L., Viegas M.F., da Silva S.L., Soares A.M., Ramos M.J., Fernandes P.A. (2022). The chemistry of snake venom and its medicinal potential. Nat. Rev. Chem..

